# Cell-Autonomous and Non-Cell-Autonomous Regulation of a Feeding State-Dependent Chemoreceptor Gene via MEF-2 and bHLH Transcription Factors

**DOI:** 10.1371/journal.pgen.1006237

**Published:** 2016-08-03

**Authors:** Matthew Gruner, Jeremy Grubbs, Aja McDonagh, Dominic Valdes, Ari Winbush, Alexander M. van der Linden

**Affiliations:** Department of Biology, University of Nevada, Reno, Reno, Nevada, United States of America; University of California San Francisco, UNITED STATES

## Abstract

Food and feeding-state dependent changes in chemoreceptor gene expression may allow *Caenorhabditis elegans* to modify their chemosensory behavior, but the mechanisms essential for these expression changes remain poorly characterized. We had previously shown that expression of a feeding state-dependent chemoreceptor gene, *srh-234*, in the ADL sensory neuron of *C*. *elegans* is regulated via the MEF-2 transcription factor. Here, we show that MEF-2 acts together with basic helix-loop-helix (bHLH) transcription factors to regulate *srh-234* expression as a function of feeding state. We identify a *cis*-regulatory MEF2 binding site that is necessary and sufficient for the starvation-induced down regulation of *srh-234* expression, while an E-box site known to bind bHLH factors is required to drive *srh-234* expression in ADL. We show that HLH-2 (E/Daughterless), HLH-3 and HLH-4 (Achaete-scute homologs) act in ADL neurons to regulate *srh-234* expression. We further demonstrate that the expression levels of *srh-234* in ADL neurons are regulated remotely by MXL-3 (Max-like 3 homolog) and HLH-30 (TFEB ortholog) acting in the intestine, which is dependent on insulin signaling functioning specifically in ADL neurons. We also show that this intestine-to-neuron feeding-state regulation of *srh-234* involves a subset of insulin-like peptides. These results combined suggest that chemoreceptor gene expression is regulated by both cell-autonomous and non-cell-autonomous transcriptional mechanisms mediated by MEF2 and bHLH factors, which may allow animals to fine-tune their chemosensory responses in response to changes in their feeding state.

## Author Summary

Plasticity in chemoreceptor gene expression may be a simple strategy by which an animal can modulate its chemosensory responses in changing external and internal state conditions. However, the transcriptional mechanisms required for these chemoreceptor gene expression changes are poorly understood. Here, we describe the identification of a transcriptional module(s) consisting of MEF-2 and basic helix-loop-helix (bHLH) transcription factors and their cognate binding sites in *Caenorhabditis elegans* that act together in ADL sensory neurons to properly regulate expression of a feeding-state dependent chemoreceptor gene. We also showed that chemoreceptor gene expression in ADL neurons are regulated remotely by bHLH factors acting in the intestine through an insulin-mediated signaling pathway, implying a sensory neuron-gut interaction for modulating chemoreceptor gene expression as a function of feeding state. This work describes transcriptional mechanisms mediated by MEF-2 and bHLH factors by which the expression of individual chemoreceptor genes in *C*. *elegans* are changed in response to changes in feeding state conditions.

## Introduction

Animals modify their chemosensory behavior depending on their feeding-state, which allows them, for instance, to optimize their food-search strategy [1]. A simple strategy by which animals can rapidly alter their chemosensory behavior is by dynamically changing the gene expression levels of chemoreceptors localized in chemosensory neurons. This form of plasticity in chemoreceptor gene expression is observed across phyla [2–7], but how feeding state signals are translated into expression level changes of chemoreceptor genes is poorly understood. Transcriptional regulation of chemoreceptor genes is likely to be the primary mechanism by which certain animals can align their chemoreceptor repertoire and fine-tune their chemosensory responses with changes in their internal nutritional state and external environment such as food availability. Yet, the identity of these transcription factors and their mode of action remain largely unknown.

The nematode *Caenorhabditis elegans* provides an ideal system to identify transcriptional mechanisms underlying chemoreceptor gene regulation in specific chemosensory neurons, and has provided insight into the *cis*- and *trans*-regulatory logic for chemoreceptor gene expression. For example, the transcription factor HMBX-1, a *C*. *elegans* homolog of HMBOX1, regulates the *srsx-3* chemoreceptor gene [8], which is asymmetrically expressed in AWC sensory neurons [9]. In the AWB sensory neurons, the KIN-29 salt-inducible kinase (SIK) regulates the expression of the *str-1* chemoreceptor gene via the MADS-box transcription factor MEF-2 [10]. Moreover, promoter analysis of several AWB expressed chemoreceptors identified a shared bi-partite motif required for AWB specific expression [11]. A systematic analysis of all *srh* chemoreceptor gene family promoters found that a bi-partite E-box motif was sufficient to direct expression in ADL sensory neurons [12], suggesting that the E-box and its cognate binding proteins the basic Helix-Loop-Helix (bHLH) transcription factors may have important roles in the regulation of ADL-expressed chemoreceptor genes.

bHLH transcription factors in *C*. *elegans* as well as in other multi-cellular organisms coordinate a number of developmental events in the nervous system, such as cell-specification and the differentiation of neurons. For example, the proneural HLH-2 factor in *C*. *elegans* is the sole ortholog of E/Daughterless proteins required for neural development [13–15] and distal cell-migration [16], while the Achaete-Scute (As/Sc) family protein, HLH-3, is needed for the differentiation of the hermaphrodite-specific neurons [17]. bHLH factors can form homodimers or heterodimers through interactions with other members of the bHLH family [18,19]. A recent comprehensive study determined the dimerization, spatiotemporal expression and E-box (CANNTG) binding specificities of nearly all members of the *C*. *elegans* bHLH family. Similar to vertebrates, the E/Daughterless protein homolog, HLH-2, in *C*. *elegans* can form heterodimers with 14 other bHLH factors, including the As/Sc factor HLH-3 [13,20]. In comparison, the Max-like 3 bHLH protein MXL-3 and TFEB ortholog HLH-30 do not need bHLH partners, but likely target overlapping genes containing the same E-box sequence [20]. Indeed, MXL-3 and HLH-30 play antagonistic roles in the *C*. *elegans* intestine to regulate expression of the same lysosomal lipases *(lipl)* genes [21]. Interestingly, the expression levels of *mxl-3* and *hlh-30* are highly dependent on *C*. *elegans* feeding state conditions [21], suggesting that MXL-3 and HLH-30 act as transcriptional switches to couple an animals’ feeding state to the regulation of metabolic gene expression in intestinal cells. Thus, many of the bHLH factors in *C*. *elegans* and other organisms have well-characterized roles in processes such as neural development and metabolic gene regulation, which could overshadow their role in orchestrating state-dependent regulation of chemoreceptor genes in chemosensory neurons.

In order to better understand how feeding-state signals are translated into expression level changes of chemoreceptor genes in chemosensory neurons, we focused in this study on the candidate chemoreceptor gene, *srh-234*, which is specifically expressed in ADL sensory neurons of *C*. *elegans*. Although the chemical or subset of chemicals detected by this chemoreceptor remains to be determined, our recent work showed that the expression levels of *srh-234* are highly dependent on feeding state conditions [22]. In fed animals, *srh-234* is strongly expressed in ADL neurons, but when starved, the expression is rapidly down regulated. Multiple pathways acting in ADL sensory neurons including KIN-29 SIK [23] and the DAF-2 insulin-like receptor [24], as well as circuit inputs from the RMG interneuron mediated by the NPR-1 neuropeptide receptor [25,26] regulate expression of *srh-234* in ADL [22]. We further showed that the MEF-2 transcription factor is required to reduce *srh-234* expression during starvation [22], suggesting that MEF-2 functions in *C*. *elegans* as a feeding state-dependent transcriptional regulator for chemoreceptor genes. Interestingly, in mammals, members of the MEF2 family can interact directly with heterodimers formed between myogenic bHLH proteins and E/Daughterless proteins to cooperatively regulate muscle gene expression [27–29]. Similarly, the bHLH heterodimer consisting of the neurogenic MASH1 and E/Daughterless protein can cooperatively interact with MEF2 by allowing either type of factor to activate transcription of neuronal genes through the binding site of the other [30]. These observations raise the intriguing possibility that a similar transcriptional module consisting of MEF-2 and certain bHLH factors may operate on chemoreceptor genes in *C*. *elegans*.

Here, we show that bHLH transcription factors act together with MEF-2 to regulate expression of the chemoreceptor gene, *srh-234*, as a function of feeding state. We show that a MEF2 binding site is necessary and sufficient for starvation-induced down regulation of *srh-234* expression, while an E-box motif in close proximity of the MEF2 site is required to direct *srh-234* expression in ADL neurons. A systematic analysis of bHLH factors identified multiple *bHLH* genes as regulators of *srh-234* expression levels. We show that mutations in *hlh-2*, *hlh-3* and *mxl-3* and *hlh-4(RNAi)* treated animals strongly reduce *srh-234* expression in fed conditions, while mutations in *hlh-10* weakly reduce *srh-234* expression. In addition, we show that *hlh-30* mutants similar to *mef-2* suppress the starvation-induced down regulation of *srh-234*. We show that HLH-2, HLH-3, and HLH-4 are transiently expressed in, and act in ADL neurons to regulate *srh-234* expression, whereas the expression levels of *srh-234* in ADL is remotely regulated by MXL-3 and HLH-30 acting in the intestine. This non-cell-autonomous regulation of *srh-234* expression mediated by MXL-3/HLH-30 requires DAF-2/DAF-16 insulin signaling in ADL. We also show that a subset of insulin-like peptides (ILPs) in the intestine, as well is in other neurons, are required to modulate *srh-234* expression. Taken together, these findings suggest that MEF-2 and bHLH transcriptional factors link feeding state conditions via insulin signaling to the regulation of chemoreceptor genes in chemosensory neurons.

## Results

### MEF2 and E-box binding sites regulate expression of the state-dependent *srh-234* chemoreceptor

We previously showed that the expression levels of a candidate chemoreceptor gene, *srh-234*, in the ADL sensory neuron type is dynamically modulated by the feeding state of *C*. *elegans* [22]. *gfp* expression driven by only 165 bp upstream sequence of the *srh-234* transcriptional start site (*srh-234*p::*gfp*) is strongly expressed in ADL neurons in fed animals, but when animals were starved for long-periods of time (>6 hours), *srh-234*p::*gfp* was significantly down regulated [22]. This strongly suggests that the feeding state-dependent regulation of *srh-234* occurs at the level of transcription.

**Fig 1:**
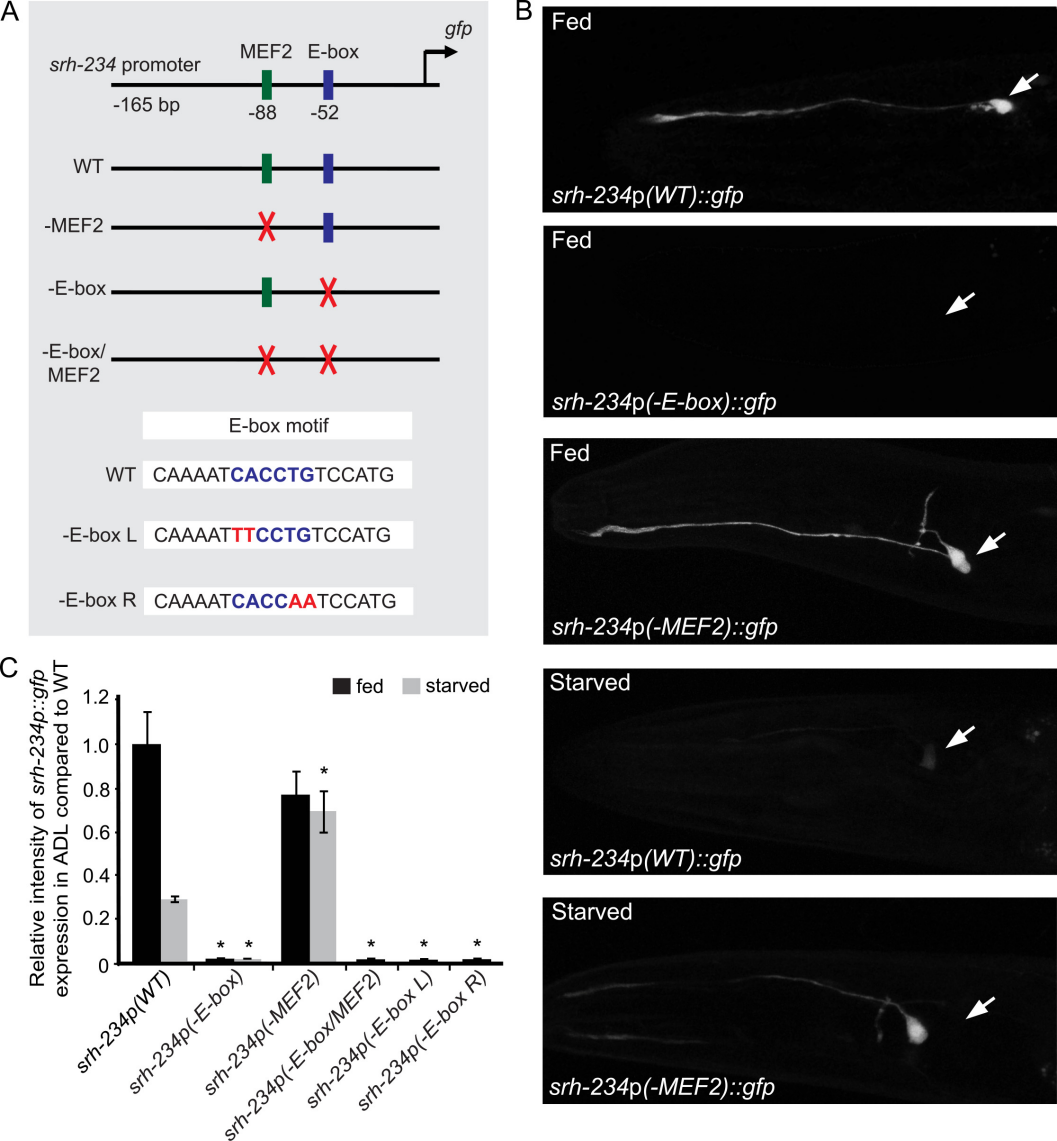
Mutagenesis of the *srh-234* regulatory region reveals an E-box and MEF2 site important for feeding-state dependent regulation of *srh-234*. **(A)** The indicated lengths and positions of predicted regulatory elements relative to the translational start site of *srh-234* fused to *gfp* in expression vectors (see Material and Methods. Sequences in green and blue indicate the predicted MEF2 and E-box sequence motifs, respectively. Mutations generated in these sequences are indicated in red. **(B)** Representative expression of ADL sensory neurons driven by wild-type and mutated *srh-234* regulatory sequences in adult wild-type animals when either well-fed in the presence of *E*. *coli* OP50 food or starved in the absence of food for 12 hours. Confocal images are lateral views of ADL sensory neurons with the arrow pointing to the cell body; anterior is left. Images were acquired at the same exposure time at room temperature. **(C)** Relative expression of mutated *srh-234* regulatory sequences in the ADL cell body compared to wild-type adults when fed or starved. Data shown is the average of least three independent transgenic lines for each genotype with n>25 adult animals for each transgenic line. * indicates values that are different from that of wild-type adult animals at *P*<0.001. Error bars denote the SEM.

To identify candidate transcription factor(s), we examined the 165 bp minimal promoter of *srh-234* using Lasagna Search 2.0 software [31], and found a predicted MEF2 factor binding site (AGTTATATTTAA, -88 bp) [32,33] and a E-box motif (CACCTG, -52 bp) known to bind bHLH transcription factors [19] in close proximity of each other (Fig 1A). To directly test whether the identified MEF2 and E-box sequence motifs are important for *srh-234* regulation, we generated *gfp*-reporter fusions of *srh-234* promoters that carry mutations in the E-box and/or MEF2 sites and monitored *gfp* expression in transgenic animals in fed and starved conditions. Mutating the E-box core of *srh-234* (-E-box; CACCTG to CTGCAG), completely abolished expression levels of *srh-234*p::*gfp* in ADL in both fed and starved conditions (Fig 1B and 1C), suggesting that the *srh-234* E-box motif is necessary to drive expression in ADL neurons. Considering that bHLH factors can bind as homo- or heterodimers to E-box motifs in *C*. *elegans* [18,19] with different bHLH family members having different preferences for the two central base pairs of the E-box [34], we also mutated either the left (*-*E-box L; CAC to TTC) or right (-E-box R; CTG to CAA) half-site of the E-box identified in the *srh-234* promoter. Similar to mutating the E-box core, mutating both the left (-E-box L) and right E-box half site (*-*E-box R) abolished expression levels of *srh-234*p::*gfp* in ADL neurons (Fig 1C). Thus, each E-box half-site is required for *srh-234* expression in ADL neurons.

To further examine whether the *srh-234* E-box motif is sufficient for expression in ADL neurons, we inserted the entire E-box sequence motif of *srh-234* with a 3-bp flanking sequence (AATCACCTGTCC) into the upstream sequence of the AWA-expressed *odr-10* chemoreceptor gene [35]. Animals carrying an *odr-10*p*(+E-box)*::*gfp* transgene showed expression of *odr-10* in ADL neurons in addition to expression in AWA neurons, albeit weaker than AWA neurons (S1 Fig). It is possible that the relevant location of the E-box insert or additional DNA flanking of the E-box site is important to properly express *odr-10* in ADL. Nevertheless, our finding is consistent with previous work showing that the E-box appears in ADL-expressed genes and functions as a potential ADL enhancer element [12]. Together, these results suggest that the *srh-234* E-box motif is both necessary and able to direct *srh-234* expression in ADL neurons.

We next mutated the MEF2 site alone or together with the E-box motif (-E-box/MEF2), and again monitored *srh-234*p::*gfp* expression levels in both fed and starved conditions. Consistent with *mef-2* mutants partially rescuing the starvation-induced reduction of *srh-234*p::*gfp* expression [22], we found that mutating the core of the MEF2 site (-MEF2; AGTTATATTTAA to AGTCGACTTTAA; Fig 1A) in the *srh-234* promoter resulted in increased *srh-234*p::*gfp* expression levels during starvation, while in fed conditions the *srh-234*p::*gfp* expression levels are not significantly reduced (Fig 1B and 1C). Mutating the E-box and MEF2 motifs together (-E-box/MEF2) again abolished *srh-234*p::*gfp* expression similar to mutating the E-box core alone (-E-box; Fig 1C). These results suggest that the identified MEF2 binding site in the 165 bp upstream sequence of *srh-234* is required to repress, but not to promote *srh-234* expression in ADL during starved conditions.

**Fig 2:**
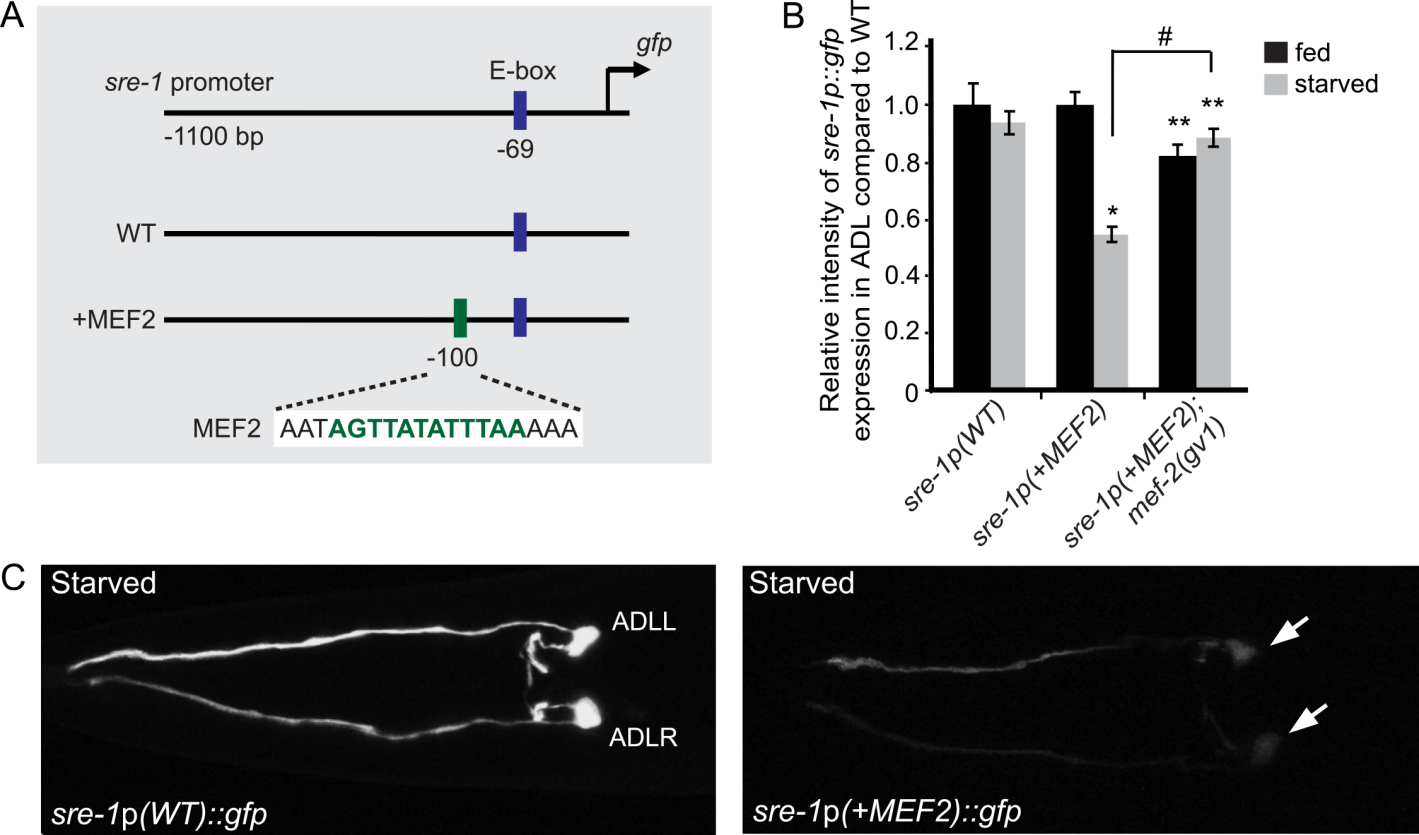
Introduction of the *srh-234* MEF2 site into the ADL-expressed *sre-1* promoter confers regulation by starvation. **(A)** The indicated lengths and positions of predicted regulatory elements relative to the translational start site of *sre-1* fused to *gfp* in expression vectors (see Material and Methods). Sequences in blue and green indicate the predicted E-box motif of *sre-1* with the inserted MEF2 site sequence of *srh-234*, respectively. **(B)** Relative expression of *sre-1* regulatory sequences in the ADL cell body without (*sre-1*p*(WT)*::*gfp*) or with the MEF2 motif of *srh-234* (*sre-1*p*(+MEF2)*::*gfp*) compared to wild-type adults when fed or starved. Data shown is the average of least two independent transgenic lines for each genotype with n>25 adult animals for each transgenic line. * indicates values that are different from that of wild-type adult animals at *P*<0.001, and # between the genotypes compared by brackets at *P*<0.001. Error bars denote the SEM. **(C)** Representative expression of ADL sensory neurons driven by wild-type *sre-1* regulatory sequences without (*sre-1*p*(WT)*::*gfp*) or with the MEF2 motif of *srh-234* (*sre-1*p*(+MEF2)*::*gfp*) in adult wild-type animals when either well-fed in the presence of *E*. *coli* OP50 food or starved in the absence of food for 12 hours. Confocal images are ventral views of ADL sensory neurons with the arrow pointing to the left and right cell body (anterior is left), and were acquired at the same exposure time at room temperature.

To further examine whether the MEF-2 site is sufficient for starvation-induced down regulation of *srh-234* expression, we inserted the MEF2 site into the upstream regulatory sequence of the ADL-expressed chemoreceptor, *sre-1*, which is not regulated by feeding state conditions [22]. We generated reporters of the *sre-1* promoter fused to *gfp* either with or without the *srh-234* MEF2 site inserted upstream in close proximity of the predicted E-box of *sre-1* (CACCTG, -69 bp; Fig 2A), and examined these transgenic animals in both fed and starved conditions. Of note, the *sre-1* promoter does not contain a predicted MEF2 binding site. We found that animals carrying the *sre-1*p*(+MEF2)*::*gfp* transgene reduced *sre-1* expression levels in ADL in starved conditions compared to wild-type animals (Fig 2B and 2C). This starvation modulation is dependent on MEF-2 as *mef-2* loss-of-function mutants carrying the *sre-1*p*(+MEF2)*::*gfp* transgene increased *sre-1* expression in starved ADL neurons (Fig 2C). Taken together, these results suggest that MEF-2 function and its candidate MEF2 binding site represses *srh-234* expression when starved, while the predicted E-box motif directs expression of *srh-234* expression in ADL.

### bHLH factors regulate the expression of *srh-234*

**Fig 3:**
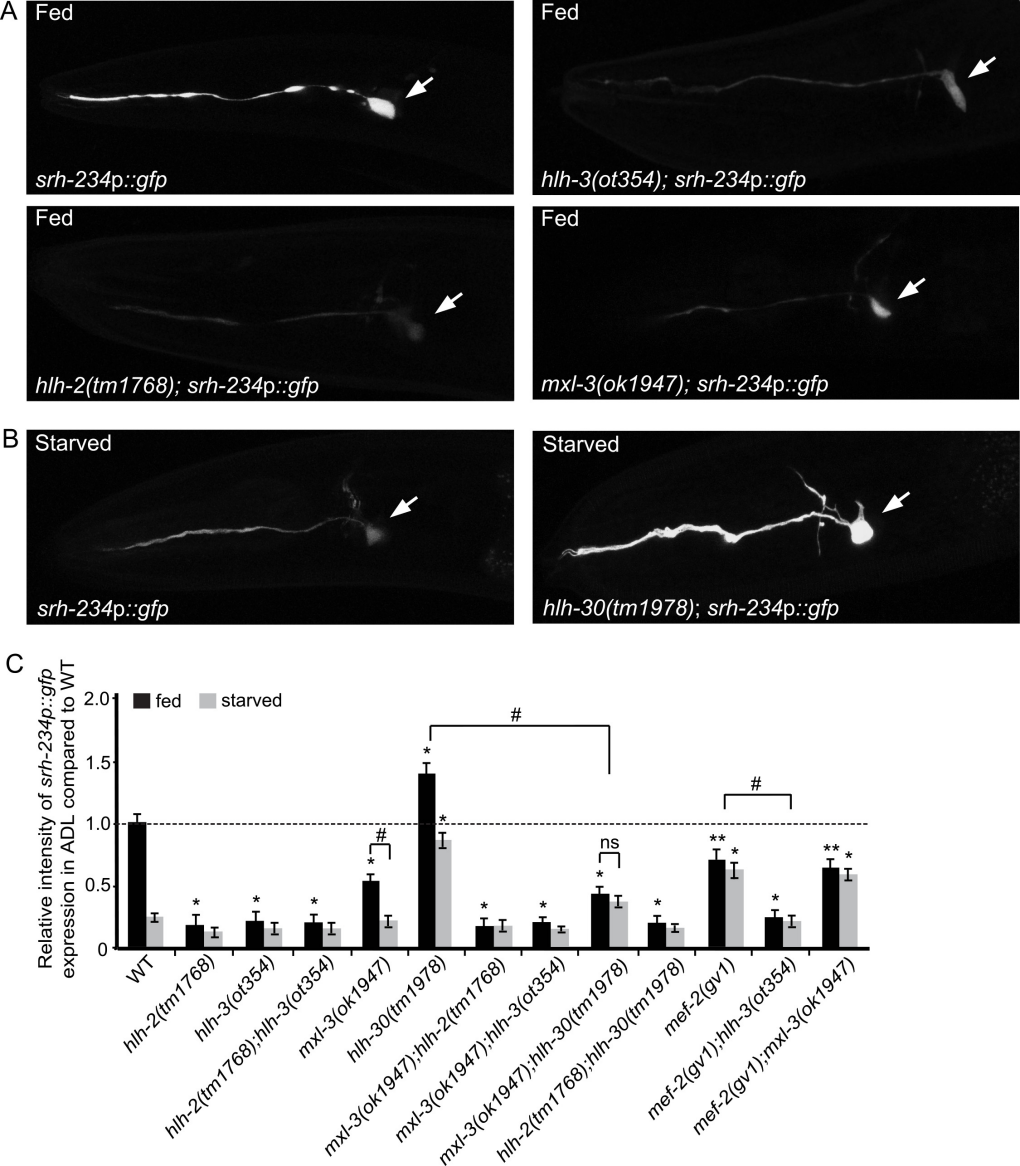
Mutations in *bHLH* genes and *mef-2* alter *srh-234* expression levels in ADL. **(A, B)** Representative expression of ADL sensory neurons driven by wild-type *srh-234* regulatory sequences fused to *gfp* (*srh-234*p::*gfp*) in adult wild-type and mutant animals of the indicated *bHLH* genes when either well-fed **(A)** in the presence of *E*. *coli* OP50 food, or starved **(B)** in the absence of food for 12 hours. Confocal images are lateral views of ADL sensory neurons with the arrow pointing to the cell body; anterior is left. Images were acquired at the same exposure time at room temperature. **(C)** Relative expression of *srh-234*p::*gfp* in adult single and double mutants of the indicated genotypes in well-fed and starved conditions. Adult animals (n>20) for each genotype were examined at the same exposure time. * and ** indicates values that are different from that of wild type adult animals at *P*<0.001 and *P*<0.01, respectively. # and ## between the genotypes compared by brackets at *P*<0.001 and *P*<0.01, respectively. Error bars denote the SEM. ns indicates not significant. All strains contain stably integrated copies of a *srh-234*p::*gfp* transgene (*oyIs56*).

Transcription factors of the bHLH protein family recognize DNA binding sites containing the E-box motif (CANNTG) [36]. The *C*. *elegans* genome contains at least 42 bHLH factors [37] that can bind E-box and/or E-box-like sequences as obligatory homo- or heterodimers with different DNA binding specificity [19,20]. To identify bHLH factors that regulate *srh-234* expression in ADL neurons, we examined *srh-234*p::*gfp* expression levels in existing mutant animals or via RNAi-mediated interference (RNAi) of *bHLH* genes in both fed and starved conditions. In particular, we focused on bHLH factors that are known or predicted to bind the E-box identified in the *srh-234* promoter. Of the 18 *bHLH* genes examined, we found that *hlh-2*, *hlh-3* and *mxl-3* mutants strongly reduced but did not abolish *srh-234*p::*gfp* expression levels in ADL during well-fed conditions, while *hlh-6* and *hlh-10* mutants and *hlh-4(RNAi)* treated animals reduced *srh-234*p::*gfp* expression (Figs 3A, 3C and S2). We further confirmed the reduced *srh-234* expression levels observed in *mxl-3* mutants by qRT-PCR (S2 Fig). Since we previously showed that ADL sensory inputs are required for *srh-234* expression [22], it is possible that the reduced *srh-234*p::*gfp* expression levels in ADL in these *bHLH* mutants results from altered sensory cilia of ADL neurons. However, with the exception of *hlh-6* mutants (30% of animals dye-fill ADL, n = 20), ADL neurons in *hlh-2*, *hlh-3*, *hlh-10*, and *mxl-3* mutants, as well as in *hlh-4(RNAi)* treated animals show wild-type dye-filling (S2 Fig), ruling out a cilia defect of ADL neurons in these mutants. Moreover, as a measure of functional integrity of ADL neurons, *hlh-2* and *hlh-3* mutants show normal avoidance responses to known chemicals sensed by ADL (e.g. CuCl_2_, glycerol, SDS and octanol) when compared to wild-type (S3 Fig).

The E/Daughterless bHLH ortholog HLH-2 is essential for early neural development [13], and can form heterodimers with 14 other bHLH factors, including HLH-3, HLH-4 and HLH-10 [20], through an E-box sequence motif (CACCTG) identical to the identified E-box motif in the *srh-234 cis*-regulatory region. However, although mutations in the *srh-234* E-box motif fully abolished expression of *srh-234*p::*gfp* in ADL neurons (Fig 1B and 1C), animals homozygous for *hlh-2(tm1768*), a hypomorphic allele that is fully sterile at 25°C and partially sterile at 20°C [16], or *hlh-3(ot354)* null mutants alone, did not abolish expression of *srh-234*p::*gfp* expression (Fig 3A and 3C). Similarly, *hlh-2(tm1768); hlh-3(ot354)* double mutants reduced but did not abolish *srh-234*p::*gfp* expression in ADL neurons at 25°C (Fig 3C). Moreover, as *hlh-2(RNAi)* results in embryonic lethality [13], we down regulated *hlh-2* selectively in ADL neurons by feeding *hlh-2(RNAi)* in *sid-1(pk3321)*; *srh-234*p::*gfp* animals carrying an *ADL*::*sid-1* transgene using the neuron-specific feeding RNAi approach [38]. Using this method, we found that *hlh-2(RNAi)* selectively in ADL neurons again reduced but did not abolish *srh-234*p::*gfp* expression levels (S7 Fig). Effects of *hlh-2* on neuronal identity of ADL can be ruled out in these RNAi experiments, because expression of the ADL-specific *srh-234* is not completely abolished in ADL and also these neurons maintain their specific ADL morphology (e.g. cilia, axon, cell-body). Thus, although it remains possible that *hlh-2(RNAi)* does not eliminate all HLH-2 activity in ADL, our results suggest that HLH-2 and HLH-3 are likely needed for proper expression of *srh-234* in ADL.

We next asked whether HLH-2 and HLH-3 regulate additional ADL-expressed chemoreceptor genes. The food-independent *sre-1* promoter is specifically expressed in ADL and contains a predicted E-box identical to the *srh-234* E-box (CACCTG), but its promoter sequence does not seem to contain a candidate MEF2 binding site. We found that *hlh-2(tm1768)* and *hlh-3(ot354)* singles as well as *hlh-2(tm1768);hlh-3(ot354)* double mutants do not reduce the expression levels of *sre-1*p::*gfp* (S4 Fig), suggesting that HLH-2 and HLH-3 do not regulate *sre-1* expression, even though both the *cis*-regulatory regions of *srh-234* and *sre-1* contain the same E-boxes. It is possible that DNA sequences flanking the E-box [39,40], or the MEF2 binding site known to function in the recruitment of bHLH factors to E-box-dependent genes [28,30], play an important role in binding specificity of bHLH factors. When we examined expression of *sre-1*p*(+MEF2)*::*gfp* in either *hlh-2* or *hlh-3* mutants, the expression in ADL was not reduced (S4 Fig). These results suggest that the introduction of the identified MEF2 site into the *sre-1* promoter sequence does not confer HLH-2 and HLH-3-mediated regulation. However, when we examined expression of *odr-10*p*(+srh-234 E-box)*::*gfp* (containing the *srh-234* E-box plus direct flanking DNA sequences; S1 Fig) in ADL in *hlh-2* mutants, we find that ADL expression is reduced, albeit weakly, suggesting that the *srh-234* E-box and direct DNA flanking sequence is likely important for HLH-2-mediated regulation.

In addition to feeding conditions, we examined whether each of these bHLH factors also regulate *srh-234*p::*gfp* expression levels in starved conditions. We found that *hlh-30* mutants, and to a lesser extent *hlh-34* mutants, suppressed the starvation-induced down regulation of *srh-234*p::*gfp* similar to *mef-2* mutants, while in fed conditions *hlh-30* and *hlh-34* mutants resulted in increased expression levels of *srh-234*p::*gfp* (Figs 3B, 3C and S2). Interestingly, *hlh-30* is known to regulate the expression of the same target genes as *mxl-3* in an antagonistic manner [21]. Our results suggest a similar antagonistic role for *mxl-3* and *hlh-30* in regulating gene expression such that *mxl-3* mutants reduce *srh-234*p::*gfp* expression when fed, while *hlh-30* mutants increase *srh-234*p::*gfp* expression when starved. In contrast, *mxl-3* and *hlh-30* mutants do not alter expression levels of the food-independent chemoreceptor gene, *sre-1* (S4 Fig), consistent with the model that HLH-30 and MXL-3 are required to regulate *srh-234* expression levels as a function of feeding state. Taken together, these results suggest that HLH-2 may partner with HLH-3 and HLH-4 to promote *srh-234* expression in ADL via the E-box, while MXL-3 and HLH-30 are necessary for state-dependent regulation of *srh-234*.

### MEF-2-mediated regulation of *srh-234* is dependent on bHLH function

bHLH proteins can interact with members of the MEF2 family to cooperatively regulate gene expression in muscle and neuronal cells [27–30]. Having implicated a candidate MEF2 binding site as well as loss of MEF-2 function in the starvation-induced down regulation of *srh-234* expression in ADL [22], we examined the genetic interactions between *mef-2* and *bHLH* genes on the regulation of *srh-234*. When we combined loss-of-function mutations in *mef-2* with *hlh-3*, the double mutants showed a reduced *srh-234*p::*gfp* expression phenotype in fed conditions similar to single *hlh-3* mutants (Fig 3C), suggesting that *hlh-3* acts downstream of, or in parallel to, *mef-2* to regulate *srh-234* expression. However, when we combined mutations in *mef-2* with *mxl-3*, double mutants exhibited expression levels of *srh-234*p::*gfp* similar to *mef-2* single mutants (Fig 3C), suggesting that *mef-2* acts genetically downstream of *mxl-3* in regulating *srh-234* in ADL. Thus, MEF-2-mediated regulation of *srh-234* expression levels in ADL is dependent on bHLH function. Combining mutations in *mxl-3* or *hlh-30* with either *hlh-2* or *hlh-3*, these double mutants showed a reduced *srh-234*p::*gfp* expression phenotype not significantly different from *hlh-2* or *hlh-3* single mutants (Fig 3C), suggesting that *hlh-2* and *hlh-3* act downstream of, or in parallel to, *mxl-3* and *hlh-30* in the regulation of *srh-234* expression. Double mutants between *mxl-3* and *hlh-30* showed a similar reduced *srh-234*p::*gfp* expression phenotype as single *mxl-3* mutants alone (Fig 3C), suggesting that *mxl-3* acts genetically downstream of, or in parallel to, *hlh-30* to regulate *srh-234*p::*gfp* expression. These results suggest that HLH-30 and MXL-3-mediated regulation of *srh-234* expression is dependent on HLH-2 and HLH-3 function.

### HLH-2/3/4 act in ADL neurons to regulate *srh-234* expression

**Fig 4:**
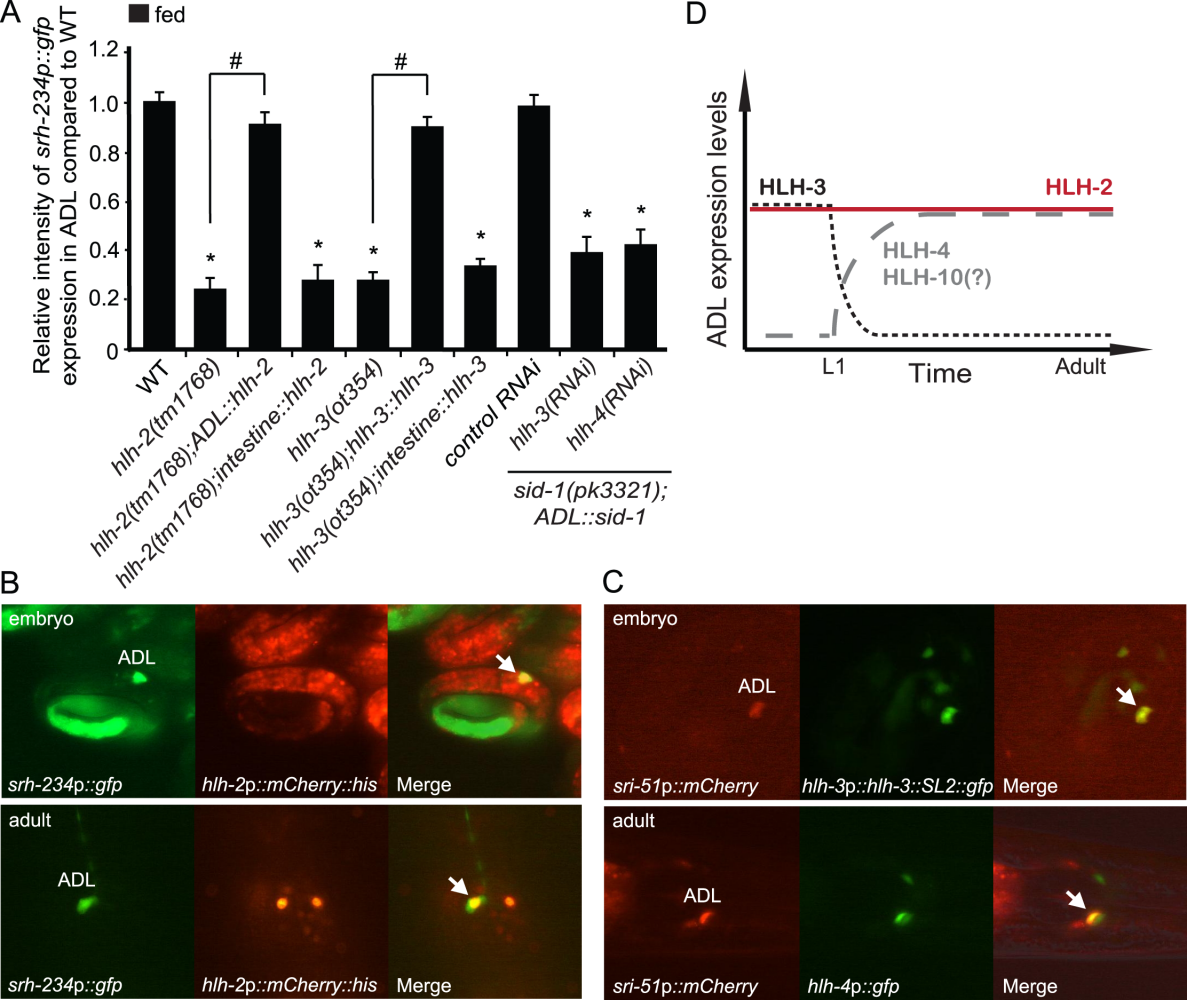
*hlh-2*, *hlh-3* and *hlh-4* act in ADL neurons to regulate *srh-234* expression, and *hlh-3* and *hlh-4* appear to be transiently expressed in ADL. **(A)** Relative expression of *srh-234*p::*gfp* in adult *hlh-2* and *hlh-3* mutant animals compared to adult wild-type, or when fed *hlh-3(RNAi)* or *hlh-4(RNAi)* compared to control RNAi. Feeding RNAi was performed in *sid-1(pk3321)him-5(e1409)* mutants with the stably integrated *oyIs56* transgene carrying a *ADL*::*sid-1* extra chromosomal transgene to selectively enhance RNAi in ADL sensory neurons but not in other tissues (see Material and Methods). Data shown is the average of at least two independent transgenic lines with n>25 adult animals for each line. * indicates values that are different from that of wild-type adult animals at *P*<0.001, and # between the genotypes compared by brackets at *P*<0.001. Error bars denote the SEM. **(B)** Expression of *hlh-2* with the ADL-specific reporter *srh-234*p::*gfp* in late embryos and in adult animals. Animals carrying the *hlh-2p*::*mCherry*::*his-11* transgene expresses *hlh-2* in the nucleus of many cells, including ADL neurons. Co-expression in ADL is indicated with arrows, and ADL appears with a green cytoplasm and yellow nucleus in a merged fluorescent image. **(C)** Expression of *hlh-3p*::*hlh-3*::*SL2*::*gfp* or *hlh-4p*::*gfp* with the ADL-specific reporter *sri-51*p::*mCherry* in embryos or adult animals. Co-expression in ADL is indicated with arrows and yellow cell in a merged fluorescent image. **(D)** Overview of the temporal expression of HLH-2/3/4/10 in ADL sensory neurons.

Heterodimers of HLH-2/HLH-3 and HLH-2/HLH-4 are known to physically bind an E-box sequence motif (CACCTG) [20], which is identical to the E-box identified in the 165 bp *cis*-regulatory region of *srh-234*. This suggests that HLH-2 and its partners HLH-3 and HLH-4 might directly modulate expression levels of *srh-234* in ADL neurons. We therefore determined whether HLH-2 function in ADL was sufficient for *srh-234* regulation by selectively rescuing the expression of *hlh-2* in ADL in a *hlh-2* mutant background, and measured its effect on *srh-234* expression in fed conditions. For this, we used the promoter of *svh-1* encoding a growth factor whose ADL-specific expression [41] is not reduced in hypomorphic *hlh-2(tm1768)* mutants (S5 Fig). We found that specific expression of *hlh-2* in ADL (*ADL*::*hlh-2*) fully restored the reduced *srh-234*p::*gfp* expression phenotype of *hlh-2(tm1768)* mutants, whereas expression in the intestine (*intestine*::*hlh-2*) did not result in a rescue (Fig 4A). These results suggest that HLH-2 function is necessary in ADL to regulate *srh-234* expression.

We next determined whether HLH-3 and HLH-4 are also required in ADL neurons to regulate *srh-234* expression. We decided again to use the neuron-specific feeding RNAi approach [38] to specifically down regulate *hlh-3* as well as *hlh-4* in ADL. We found that feeding either *hlh-3(RNAi)* or *hlh-4(RNAi)* in *sid-1(pk3321)* mutant animals carrying both a *ADL*::*sid-1* transgene and a *srh-234*p::*gfp* transgene (*oyIs56*) reduced *srh-234* expression levels in ADL during feeding conditions compared to control animals (Figs 4B and S7). In addition, expression of *hlh-3* under control of its endogenous promoter (*hlh-3*::*hlh-3*), but not in the intestine (*intestine*::*hlh-3*), fully restored the reduced *srh-234* expression phenotype of *hlh-3(ot354)* null mutants (Fig 4A). Collectively, these results suggest that the function of HLH-2, HLH-3 and HLH-4 are necessary in ADL neurons to regulate *srh-234* expression.

Next, because we showed that HLH-2 and its partners HLH-3 and HLH-4 act in ADL to regulate *srh-234* expression, and because these bHLH factors are co-expressed in head neurons and other tissues in a temporal manner [20], we performed co-expression analyses to determine when they are expressed in ADL. We therefore created animals carrying either *hlh-2*p::*mCherry*::*his-11*, *hlh-4*p::*gfp*, or a rescuing *hlh-3*p::*hlh-3*::*SL2*::*gfp* construct together with an ADL-specific reporter (*srh-234*p::*gfp* or *sri-51*p::*mCherry*) (see Material and Methods), and observed that *hlh-2*, *hlh-3*, and *hlh-4* are all expressed in ADL neurons but in a temporal specific manner (Figs 4C, 4D and S6). While *hlh-2* is expressed in ADL throughout development from the embryo to the adult consistent with previous reports [13], we find that both *hlh-3* and *hlh-4* are transiently expressed in ADL (Fig 4C, 4D and 4E). Expression of *hlh-3* in ADL is observed in the late embryo before hatching, but not in L2 larvae or in adults (Figs 4D and S6). However, it remains possible that *hlh-3* is very lowly expressed in ADL. In contrast, we observed that *hlh-4* is expressed in ADL neurons in L1 larvae and in adults, but we did not observe *hlh-4* expression in ADL in late-staged embryos before hatching (Figs 4D and S6). Since HLH-4 may share target genes with HLH-10 in the same cell(s) as previously proposed [20], and because mutations in *hlh-10* reduce *srh-234*p::*gfp* expression in fed conditions, albeit weakly (S2 Fig), we also examined the expression pattern of *hlh-10*. We observed that similar to *hlh-4*, *hlh-10* is expressed in ADL neurons in larvae and adults (S6 Fig), again opposite to the expression of *hlh-3* observed in ADL. Thus, while *hlh-2* is expressed in ADL throughout development, *hlh-3* and *hlh-4* may be transiently expressed in ADL.

### MXL-3 and HLH-30 act in the intestine to remotely regulate *srh-234* expression in ADL

**Fig 5:**
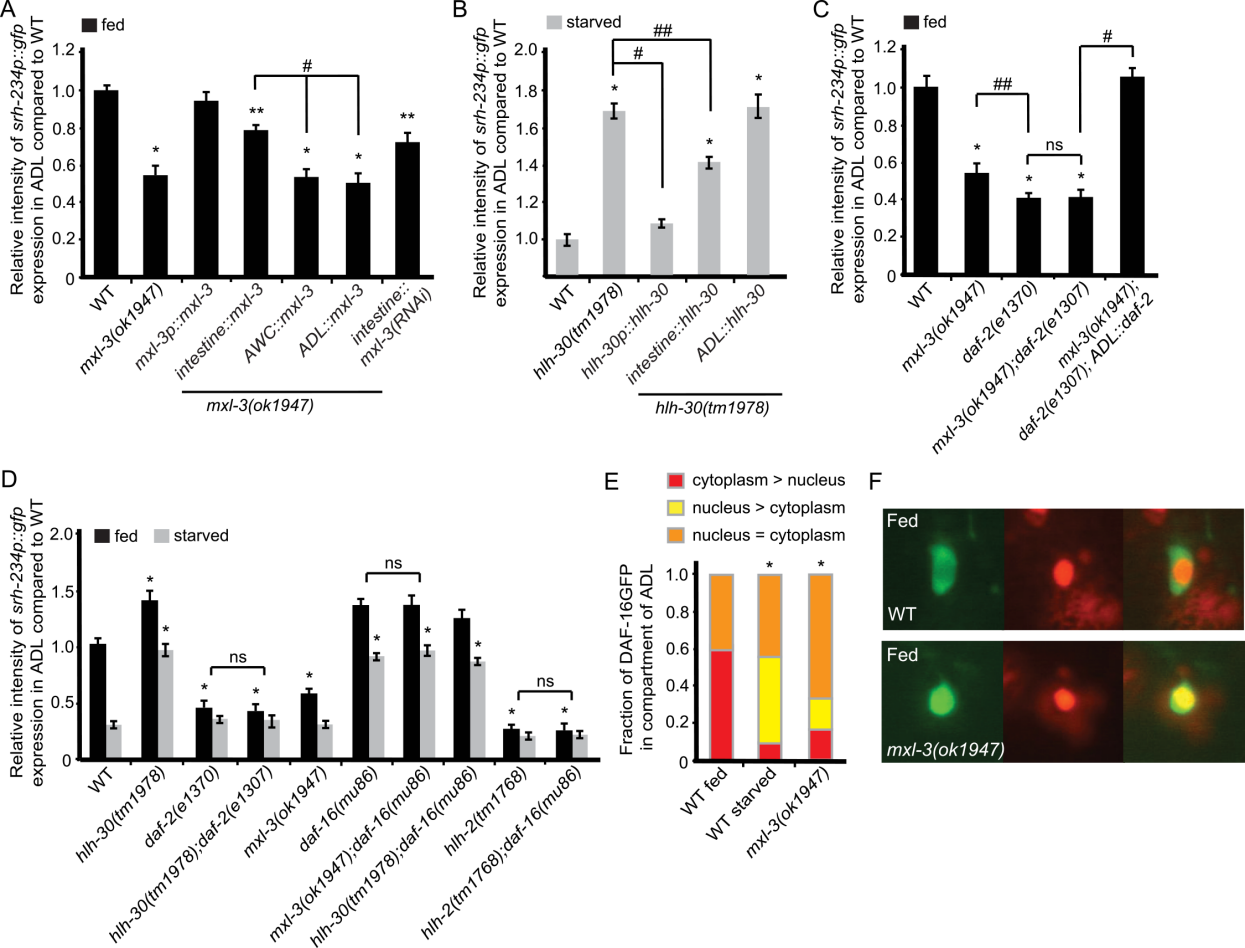
Intestinal *mxl-3* and *hlh-30* expression regulates *srh-234* in ADL neurons, which is dependent on *daf-2/daf-16* insulin signaling. **(A, B)** Selective expression of *mxl-3* and *hlh-30* in the intestine is partly sufficient to regulate *srh-234* expression in ADL. Shown is the relative expression of *srh-234*p::*gfp* in the ADL cell body of adult *mxl-3* **(A)** or *hlh-30* mutants **(B)** compared to wild-type adult animals when well-fed **(A)** and starved **(B)**, respectively. **(C)** Selective expression of the *daf-2* insulin-like receptor in ADL suppresses the reduced *srh-234* phenotype of adult *mxl-3; daf-2* double mutants. Shown is the relative expression of *srh-234*p::*gfp* in adult single and double mutants of *mxl-3* and *daf-2* compared to wild-type adults during well-fed conditions. *daf-2(e1307)* is a temperature sensitive allele. For these experiments, animals were raised at 15°C (permissive temperature) and shifted to the 25°C (restrictive temperature) as L4-staged larvae. **(D)** Mutations in *daf-2* and *daf-16* suppress the *srh-234* expression phenotypes of *hlh-30* and *mxl-3* mutants, respectively, whereas *hlh-2* suppresses *daf-16* in regulating the expression of *srh-234*. Shown is the relative expression of *srh-234*p::*gfp* in the ADL cell body in adult single and double mutants of *hlh-30*, *daf-2* or *daf-16* compared to wild-type adults in well-fed and starved conditions with >25 adult animals for each genotype. **(A-D)** data shown is the average of at least two independent transgenic lines with n>25 adult animals for each line. * and ** indicates values that are different from that of wild-type adult animals at *P*<0.001 and *P*<0.01, respectively, and # and ## between the genotypes compared by brackets at *P*<0.01 and *P*<0.05, respectively. Error bars denote the SEM. **(E, F)** Quantification **(E)** and representative images **(F)** of subcellular localization of DAF-16::GFP in ADL neurons of adult wild-type and *mxl-3* mutants in either fed or starved conditions. n>20 adult animals for each genotype and condition. * indicates values that are different from fed wild-type distribution at *P*<0.001 as determined by the χ^2^ test.

Our systematic analysis of *bHLH* loss-of-function mutants suggests that MXL-3 and HLH-30 regulate *srh-234* expression as a function of feeding state. We next determined the site(s) of action of MXL-3 and HLH-30 for regulation of *srh-234*. Expression of *mxl-3* or *hlh-30* under control of its endogenous promoter (*mxl-3*::*mxl-3* or *hlh-30*::*hlh-30*) fully rescued the reduced *srh-234* expression phenotype of *mxl-3* and *hlh-30* mutants as a function of feeding state (Fig 5A and 5B). However, when we selectively expressed *mxl-3* or *hlh-30* in ADL using the *sre-1* promoter (*ADL*::*mxl-3* or *ADL*::*hlh-30*), we did not observe any rescue of the *srh-234* expression phenotypes of *mxl-3* and *hlh-30* mutants, respectively (Fig 5A and 5B). This lack of rescue could not be attributed to MXL-3 or HLH-30 regulating *sre-1*, because mutations in *mxl-3* and *hlh-30* did not reduce expression levels of *sre-1*p::*gfp* in ADL (S4 Fig). Moreover, selective down regulation of *mxl-3* in ADL by feeding RNAi also did not change the *srh-234* expression levels (S7 Fig). Thus, MXL-3 and HLH-30 are required in cells/tissues other than ADL neurons to regulate *srh-234* expression.

Since both MXL-3 and HLH-30 act in the intestine to regulate transcription of lysosomal lipase (*lipl*) genes in response to starvation [21], we sought to determine whether MXL-3 and HLH-30 may act in the intestine to remotely regulate *srh-234* expression in ADL. Indeed, selective expression of *mxl-3* in intestinal cells using the *ges-1* promoter (*intestine*::*mxl-3*), as well as expression of *hlh-30* cDNA in the intestine (*intestine*::*hlh-30*) partially rescued the *srh-234*p::*gfp* expression phenotype of *mxl-3* and *hlh-30* mutants in fed and starved conditions, respectively (Fig 5A and 5B). Consistent with these findings, tissue-specific inactivation of *mxl-3* in the intestine using a promoter-driven hairpin RNAi strategy (Fig 5A) [42], or feeding *mxl-3(RNAi)* in *sid-1(pk3321); srh-234*p::*gfp* mutant animals carrying a *intestine*::*sid-1* transgene reduced *srh-234*p::*gfp* expression levels in ADL (S7 Fig). However, expression of *mxl-3* or *hlh-30* under control of its endogenous promoter (*mxl-3*::*mxl-*3 or *hlh-30*::*hlh-3*) fully rescued the *srh-234* expression phenotype of *mxl-3* and *hlh-30* mutants in fed and starved conditions, respectively (Fig 5A and 5B), suggesting that additional *mxl-3*- and *hlh-30*-expressed cells and/or tissues may contribute to *srh-234* regulation. Because MXL-3 is expressed in the AWC sensory neuron [21], which is a key neuron for dauer regulation as a function of feeding state [43], we tested whether *mxl-3* expression in AWC is required to regulate *srh-234*. However, expression of *mxl-3* specifically in the AWC neurons under control of the *str-148* promoter (*AWC*::*mxl-3*) did not restore the *mxl-3*-mediated reduction of *srh-234* expression in fed conditions (Fig 5A). Collectively, these results support the model that both MXL-3 and HLH-30 function is required in the intestine, at least in part, to non-cell-autonomously regulate *srh-234* expression in ADL.

### MXL-3 and HLH-30-mediated regulation of *srh-234* is dependent on insulin signaling

We next asked whether certain nutritional signaling pathways are required for this MXL3- and HLH-30-mediated regulation of *srh-234* expression in ADL. As insulin signaling is coupled to the feeding and nutritional status of many animals including *C*. *elegans* [44], and because we previously showed that the function of the DAF-2 insulin-like receptor and its downstream target DAF-16 FOXO transcription factor are specifically required in ADL to regulate *srh-234* expression [22], we asked whether MXL-3/HLH-30-mediated regulation of *srh-234* in ADL is dependent on insulin signaling. Consistent with our previous findings [22], *srh-234* expression is strongly reduced in well-fed *daf-2* mutants (Fig 5D). When we combined mutations in *daf-2* or *daf-16* with *mxl-3*, the double mutants exhibited a *srh-234*p::*gfp* expression phenotype in fed conditions similar to *daf-2* and *daf-16* single mutants, respectively (Fig 5C and 5D), suggesting that the *daf-2*/*daf-16* insulin signaling pathway acts genetically downstream of, or in parallel to *mxl-3*, to regulate *srh-234*p::*gfp* expression. Consistent with these findings, specific expression of *daf-2* cDNA in ADL neurons fully restored the reduced *srh-234*p::*gfp* expression phenotype of *mxl-3; daf-2* double mutants (Fig 5C). We also tested genetic interactions between *daf-2* and *hlh-30*, and found that mutations in *daf-2* fully suppresses the increased *srh-234*p::*gfp* expression phenotype of *hlh-30* mutants in starved conditions (Fig 5D), suggesting that *daf-2* acts downstream of *hlh-30* in *srh-234* regulation. As expected, *daf-16* acts genetically upstream of, or in parallel to, *hlh-2* in regulating *srh-234* expression as mutations in *daf-16* did not suppress the *hlh-2*-mediated reduction in *srh-234*p::*gfp* expression (Fig 5D). Thus, *daf-2/daf-16* insulin signaling pathway may act genetically upstream of *hlh-2*, but downstream of the *mxl-3* and *hlh-30* in regulating *srh-234* expression.

To further examine the connection between MXL-3 function in the intestine and the transcriptional pathways that act in ADL neurons (i.e. MEF-2, DAF-16 and HLH-2) to regulate *srh-234* [22], we first asked whether expression levels of *mef-2* are changed in response to starvation and to changes in MXL-3 and DAF-2 signaling. Interestingly, we found that both *mxl-3* and *daf-2* mutants as well as prolonged starvation increases the expression of a previously reported translational MEF-2::GFP fusion transgene [10] in many head neurons (>2.7x fold increase in *mef-2*p::*mef-2*::*gfp* expression in starved wild-type, *mxl-3* and *daf-2* mutants compared to fed wild-type animals; S8 Fig). No detectable expression changes are observed for *hlh-2* between fed and starved wild-type animals, as well as between wild-type and *daf-2* or *daf-16* mutants (S8 Fig). Thus, MEF-2 function is dependent on starvation and DAF-2 and MXL-3 signaling, consistent with our previous data showing that MEF-2 may act as a state-dependent transcriptional regulator of *srh-234* expression.

We next asked whether MXL-3 function changes the subcellular localization of DAF-16 to regulate *srh-234* expression. Previous work has shown that DAF-16::GFP shuttles between the cytoplasm and the nucleus in response to changes in environmental conditions, such as starvation and heat shock [45]. Moreover, reduced *daf-2* insulin signaling results in the nuclear localization of DAF-16::GFP [46]. If mutations in *mxl-3* reduce *srh-234* expression in fed conditions due to reduced *daf-2* signaling, we would predict that DAF-16::GFP subcellular localization in ADL neurons is similarly affected in *mxl-3* mutants by shuttling DAF-16::GFP into the nucleus of ADL. We therefore generated transgenic animals that express a DAF-16::GFP fusion protein controlled by the *sre-1* promoter (*ADL*::*daf-16*::*gfp*) in a *hlh-2p*::*mCherry*::*his* transgenic background. In these transgenic adult animals, DAF-16::GFP localizes specifically to ADL neurons with *mCherry* fluorescence in the nucleus of ADL. In fed conditions, we found that DAF-16::GFP is mostly present in the cytoplasm in wild-type animals (Fig 5E and 5F). However, after prolonged starvation, DAF-16::GFP shuttles (at least in part) to the nucleus (Fig 5E), suggesting that the DAF-16::GFP transgene appears to be functional. When we examined this transgene in a *mxl-3* mutant background, the DAF-16::GFP becomes similarly localized in the nucleus as in starved conditions (Fig 5E and 5F), although not all of the DAF-16::GFP fusion proteins are fully excluded from either the cytoplasmic or nuclear compartment, possibly due to overexpression of the DAF-16::GFP transgene in ADL neurons. These results suggest that starvation regulates DAF-16 subcellular localization in ADL, and that MXL-3 function may be important for subcellular localization of DAF-16 in ADL. Taken together, these results support the model that MXL-3 regulates both MEF-2 and DAF-16-mediated mechanisms to regulate *srh-234* expression.

### Insulin-like peptides regulate *srh-234* expression, which is dependent on MXL-3 function

**Fig 6:**
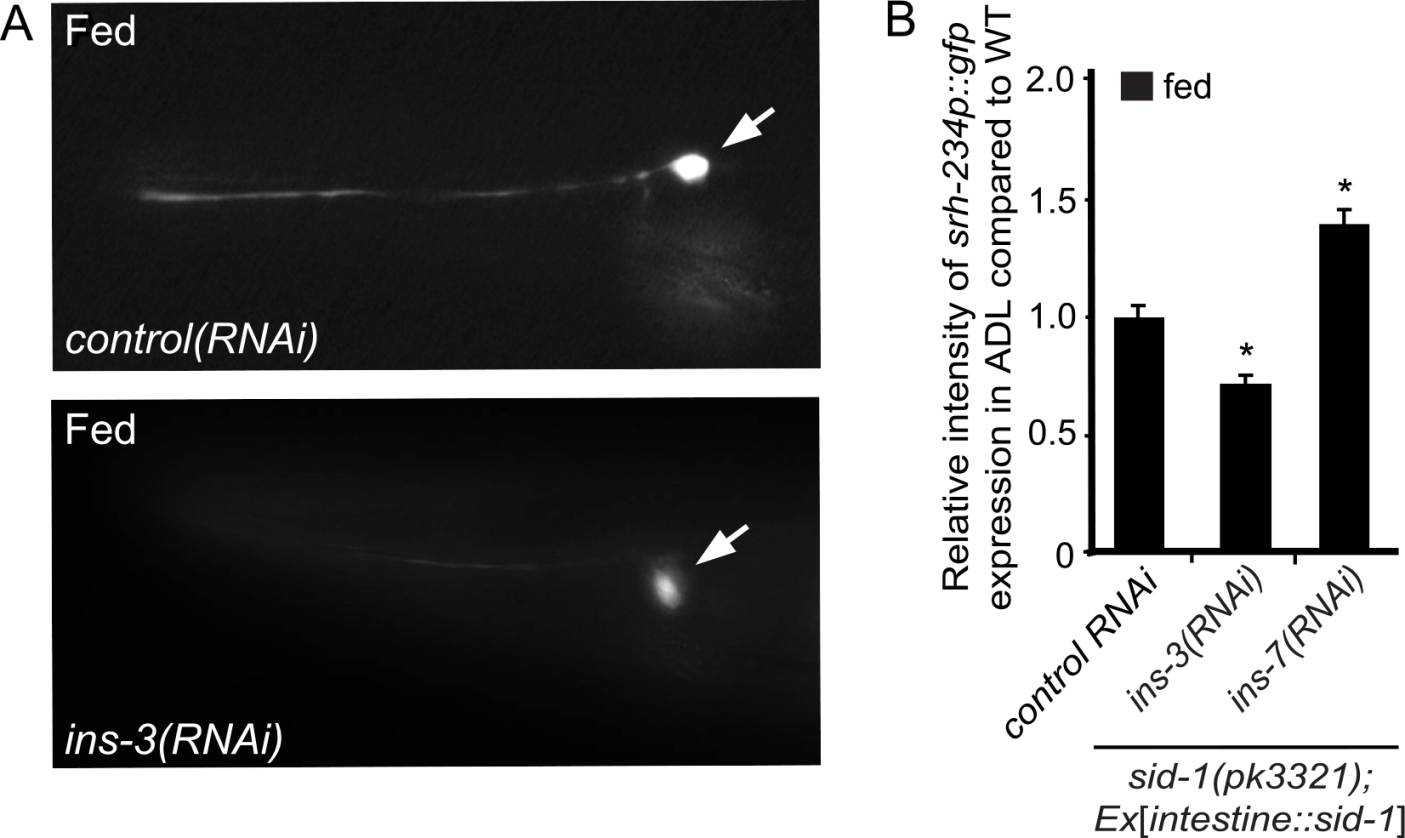
Intestine-specific RNAi knockdown of *ins-3* and *ins-7* alters *srh-234* expression levels in ADL. **(A)** Representative expression of *srh-234*p::*gfp* in adult animals after feeding either *ins-3(RNAi)* or control RNAi. Feeding RNAi was performed in *sid-1(pk3321)him-5(e1409)* mutants with the stably integrated *oyIs56* transgene carrying the *intestine*::*sid-1* extra chromosomal transgene to selectively enhance RNAi in the intestine, but not in other tissues (see Material and Methods). Images are lateral views of ADL with the arrow pointing to the cell body; anterior is left. Images were acquired at the same exposure time at room temperature. **(B)** Relative expression of *srh-234*p::*gfp* in the ADL cell body when fed either *ins-3(RNAi)* or *ins-7(RNAi)* compared to adult animals fed with control RNAi. Data shown is the average of at least two independent transgenic lines with n>20 adult animals for each line. * indicates values that are different from that of wild-type adult animals at *P*<0.001. Error bars denote the SEM.

To further test the model that MXL-3 function in the intestine acts through insulin signaling to regulate *srh-234* expression in ADL neurons, we sought to identify insulin-like peptides (ILPs) that may target the DAF-2 insulin-like receptor acting in ADL. Since a semi-dominant mutation (*sa191*) in *daf-28* that is suggested to block other agonistic ILPs for DAF-2 [47] reduces *srh-234* expression in fed conditions [22], we first tested whether DAF-28 itself may be required for *srh-234* regulation. However, *daf-28* null mutants do not appear to alter *srh-234* expression in fed or starved conditions (S9 Fig), suggesting that other ILPs are required to regulate *srh-234* expression in ADL. To identify these ILPs, we decided again to use the intestine-specific knockdown RNAi approach in the *sid-1* mutant background [38] by feeding RNAi directed against individual *ILP* genes (S9 Fig). Of the 29 *ILP* genes examined, we found that selective down regulation of *ins-3(RNAi)* in the intestine reduced *srh-234* expression in ADL, whereas *ins-7(RNAi)*, *ins-21(RNAi)*, and *ins-28(RNAi)* increased *srh-234* expression (Figs 6A, 6B and S9). Thus, selective down regulation of a subset of *ILP* genes in the intestine alters the expression of *srh-234* in ADL. Since *ins-3* and *ins-7* expression in the intestine is dependent on feeding state conditions [48,49], and because *mxl-3* transcription is down regulated by starvation [21], we next asked whether the expression of these *ILP* genes are regulated by *mxl-3*. We focused on the *ins-3* ILP because the promoter sequence of the *ins-3* gene contains candidate MXL-3 and HLH-30 sites as determined by Lasagna 2.0 software (S1 Table) [31]. Interestingly, we found that intestinal expression but not neuronal expression of *ins-3*p::*gfp* is reduced, albeit weakly, in *mxl-3(ok1947)* mutants (S10 Fig) (1.8x fold decrease of *ins-3*p::*gfp* in the intestine in *mxl-3* compared to wild-type), suggesting that MXL-3 may directly regulate *ins-3* expression in the intestine. No detectable expression differences between wild-type and *mxl-3(ok1947* mutants were observed for *ins-4*p::*gfp* and *ins-5*p::*gfp* reporters (S10 Fig). Of note, selective down regulation of *ins-4* and *ins-5* in the intestine by RNAi do not appear to alter *srh-234* expression (S9 Fig). Thus, intestinal MXL-3 regulates *srh-234* expression remotely in ADL neurons, possibly through the function of the *ins-3* ILP.

We further show that the expression of *srh-234*p::*gfp* in ADL neurons is increased in loss-of-function mutants of *ins-26* ILP in both fed and starved conditions when compared to wild-type animals (S9 Fig), while selective down regulation of *ins-26* by RNAi in the intestine does not affect *srh-234*p::*gfp* expression in ADL (S9 Fig). The *ins-26* ILP is known to be expressed in ASE, ASI and AWC sensory neurons in addition to the intestine and other cells, but is not known to be expressed in ADL [48]. Recent work indicated that the expression of *ins-26* in AWC neurons is dependent on food availability; *ins-26* expression in AWC is reduced upon starvation [43]. When we examined *ins-26*p::*gfp* expression in a *mxl-3* mutant background in fed conditions, we observed that *ins-26* expression was strongly increased in cells tentatively identified as ASE neurons (and possibly ASI) (S10 Fig). In wild-type animals, this *ins-26*p::*gfp* transgene is weakly expressed in head neurons [50]. Interestingly, intestine-specific expression of *mxl-3* (*intestine*::*mxl-3*) could rescue this observed increase of *ins-26p*::*gfp* expression in ASE in *mxl-3* mutants back to near wild-type levels (only 3% animals carrying the *intestine*::*mxl-3* transgene show strong expression of *ins-26*p::*gfp* in ASE compared to 84% animals without the transgene). Thus, MXL-3 plays a non-cell-autonomous role for regulation of *srh-234* expression levels in ADL, either by regulating *ILP* genes in the intestine (e.g. *ins-3*), or more indirectly by regulating the expression of *ILP* genes in neurons other than ADL (e.g. *ins-26*). Together, these results support the model that MXL-3 function in the intestine acts through insulin-mediated signaling to remotely regulate *srh-234* expression in ADL.

## Discussion

**Fig 7:**
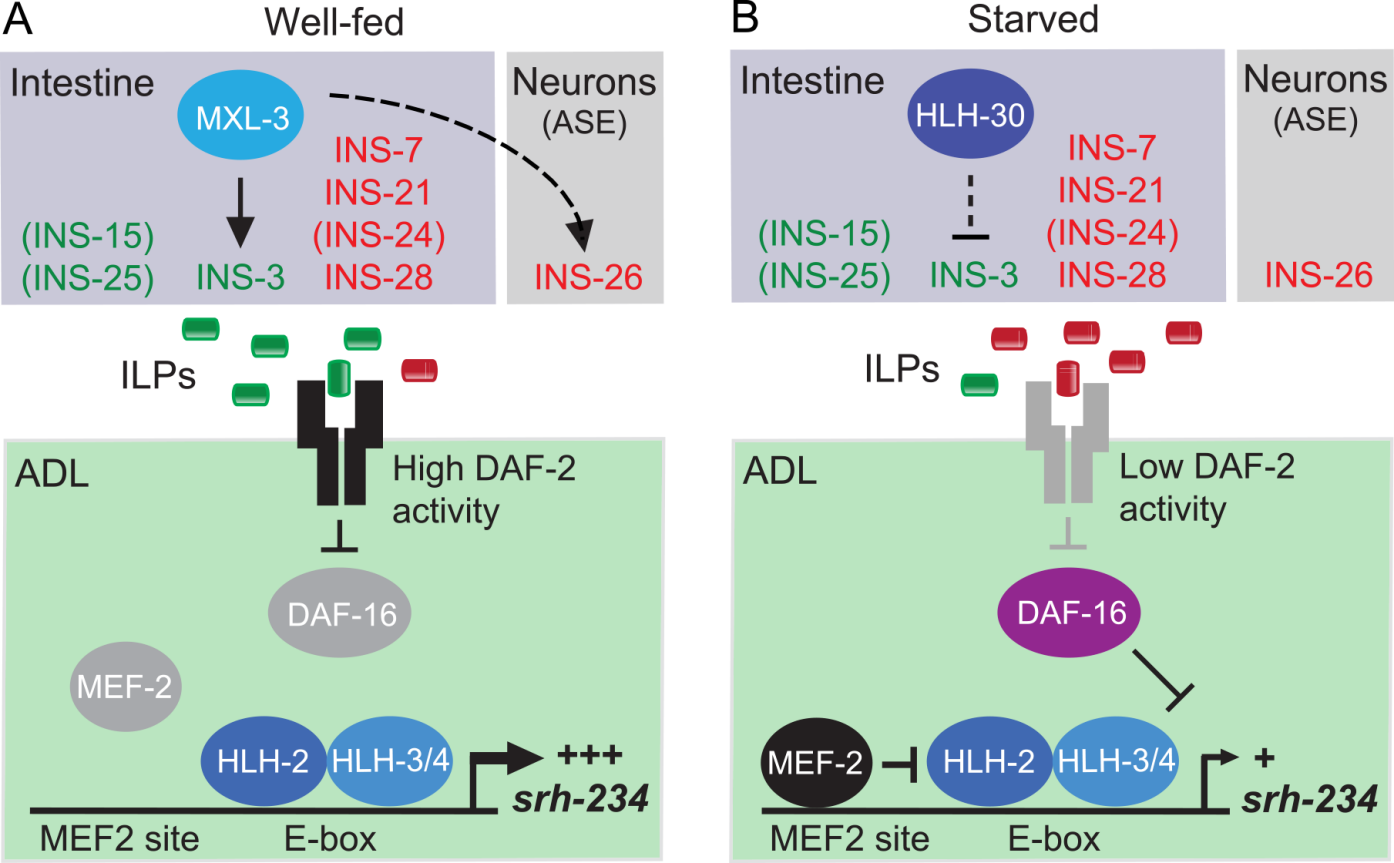
An intestine-to-neuron model for regulation of *srh-234* expression in ADL as a function of feeding state. Changes in expression of *srh-234* are directed by multiple cell-autonomous and non-cell-autonomous transcriptional modules. In ADL neurons, one module consists of a MEF-2 transcription factor and its MEF2 binding site, and the other module consists of a bHLH heterodimer (e.g. HLH-2/3, and HLH-2/4) and its bi-partite E-box binding site. When fed **(A)**, MEF-2 activity is low and is prevented from repressing HLH-2/3/4 factors, and expression of *srh-234* in ADL is increased. When starved **(B)**, MEF-2 is no longer inhibited, allowing binding to the MEF2 site in *srh-234* (AGTTATATTTAA), which in turn represses HLH-2/3/4-mediated activation of *srh-234* at the E-box site (CACCTG), ultimately leading to the decrease in *srh-234* expression. In addition to cell-autonomous mechanisms, the bHLH factors MXL-3 and HLH-30 act in the intestine to remotely regulate changes in *srh-234* expression via the DAF-2/DAF-16 insulin pathway acting in ADL. When fed **(A)**, MXL-3 activity is high in the intestine (low HLH-30 activity) and increases *srh-234* expression, whereas when starved **(B)**, HLH-30 activity is high in the intestine (low MXL-3 activity) and represses the expression of *srh-234* in ADL. The combined action of ILPs from different tissues (the intestine) and other neurons (e.g. ASE) regulated by *mxl-3*-dependent (e.g. *ins-3*, *ins-26*) or independent mechanisms results in either high or low DAF-2 activity, which in turn modulates *srh-234* expression in ADL.

In this study, we investigated the transcriptional mechanisms by which feeding-state information is translated into expression level changes of chemoreceptor genes, using the *srh-234* promoter of *C*. *elegans* as a model system. The results reveal an important role for MEF2 and basic helix-loop-helix (bHLH) transcription factors in the regulation of *srh-234* expression as a function of feeding state. We identified transcriptional module(s) consisting of MEF-2 and bHLH factors that act together in ADL neurons in a temporal manner to properly regulate *srh-234* expression. We also showed that the expression levels of *srh-234* in ADL are regulated remotely by bHLH factors acting in the intestine, through an insulin-mediated signaling pathway. These results suggest that both cell-autonomous and non-cell-autonomous transcriptional mechanisms mediated by MEF-2 and bHLH factors regulate *srh-234* gene expression levels (Fig 7), providing a sensory neuron-gut interaction for modulating chemoreceptor gene expression as a function of feeding state.

### MEF-2 and HLH-2/3/4 act cell-autonomously in ADL through MEF2 and E-box sites to regulate *srh-234* expression

Our results reveal a new role for the E/Daughterless ortholog HLH-2 and Achaete-scute homolog HLH-3 in the regulation of chemoreceptor genes in addition to their well-characterized function in nervous system development, such as in the establishment of neuronal cell fates [13–17]. HLH-2 is known to form heterodimers with both HLH-3 and HLH-4 *in vivo* [13,20], and we find that they all act in ADL sensory neurons to similarly reduce *srh-234* expression levels in fed conditions. While *hlh-2* is expressed throughout development in ADL neurons, *hlh-3* and *hlh-4* appear to be transiently expressed in ADL, with *hlh-3* being expressed in ADL in early embryo but not in adults, while *hlh-4* is expressed in ADL in adults but not in embryo. If *hlh-3* is not expressed in adult ADL neurons, then how can *hlh-3* regulate *srh-234* expression in the adult? It is possible that *hlh-3* is important to first initiate *srh-234* expression in ADL in the early embryo, such that the following sequential steps to maintain *srh-234* expression into adulthood do not occur. These later steps could involve *hlh-4* and/or *hlh-10*, or alternatively *hlh-3* may regulate other signaling genes required to maintain *srh-234* expression through development. Although the exact mechanisms underlying *srh-234* regulation mediated by these bHLH factors in ADL neurons is not yet clear, we speculate that *srh-234* may be regulated by a combination of different bHLH heterodimer pairs in a temporal-specific manner. Switching has been shown to occur among heterocomplexes of bHLH factors. For instance, the switch from Myc:Max to Mad:Max bHLH pairs whom all recognize the same E-box motif results in a change in transcriptional regulation of target genes required for cell proliferation [51]. A similar switching mechanism may operate between bHLH pairs in *C*. *elegans* in order to properly initiate and maintain expression of *srh-234* in ADL neurons throughout development.

Mutational analysis of the *cis*-regulatory region of *srh-234* identified an E-box site known to physically bind bHLH heterodimers, such as HLH-2/3 and HLH-2/4 [13,20]. In *C*. *elegans*, the E-box is highly enriched in the *srh*-family of chemoreceptor genes, and is able to drive expression in ADL neurons [12]. Consistent with these findings, introduction of the *srh-234* E-box motif in the regulatory sequence of the AWA-expressed *odr-10* chemoreceptor gene resulted in *odr-10* expression in both AWA and ADL neurons. Thus, the identified E-box in the *srh-234* regulatory sequence is able to direct expression in ADL. However, we did not detect any requirement for HLH-2 and HLH-3 in directing the expression of *srh-234* in ADL neurons, such that levels of *srh-234* expression are not fully abolished in single or double mutants between *hlh-2* and *hlh-3*, and/or via ADL-specific down regulation of these *bHLH* genes by feeding RNAi. Thus, although residual activity of bHLHs cannot be ruled out in these experiments, we suggest that additional factors are required to initiate expression of *srh-234* in ADL neurons.

In contrast to *srh-234* gene regulation, we find that mutations in *hlh-2* and *hlh-3* do not alter the expression levels of a food-independent chemoreceptor, *sre-1*, in ADL. Thus, HLH-2 and HLH-3 do not appear to regulate additional ADL-expressed chemoreceptors, even though the core of the predicted *sre-1* E-box is identical to the *srh-234* E-box (CACCTG). Nucleotide sequences directly flanking the core E-box motif are known to influence the DNA binding specificity and affinity of bHLH factors [39,40], which could partly explain the expression differences observed for *sre-1* and *srh-234* in *hlh-2* and *hlh-3* mutants. Consistent with these observations, expression of *odr-10*p*(+srh-234 E-box)*::*gfp* in ADL neurons in *hlh-2* mutations is reduced, suggesting that the *srh-234* E-box site and direct flanking sequence seems to play an important role in HLH-2-mediated regulation.

In addition to the *srh-234* E-box, we show that the *cis*-regulatory sequence of *srh-234*, but not that of *sre-1*, contains a putative MEF2 binding site in close proximity to its E-box. We find that this MEF2 site is necessary to modulate *srh-234* expression levels upon starvation. However, MEF-2 does not itself dictate expression in ADL neurons, as loss-of-function mutations in *mef-2* or mutation of its putative MEF2 binding site did not reduce *srh-234* expression in ADL in fed conditions [22]. Thus, the MEF-2 transcription factor acts to repress *srh-234* expression when starved, but it is likely not required to promote *srh-234* expression when fed. Intriguingly, introduction of the *srh-234* MEF2 site into the regulatory sequence of *sre-1* close to its predicted E-box motif (i.e. *sre-1*p*(+MEF2)*::*gfp*) was sufficient to confer starvation-induced down regulation via MEF-2. It is known that bHLH factors can be recruited to activate E-box-dependent genes when they also contain a MEF2 binding site [28,30]. However, when we examined expression of *sre-1*p*(+MEF2)*::*gfp* in either *hlh-2* or *hlh-3* mutants, the *sre-1* expression in ADL was not reduced. These results suggest that introduction of a MEF2 binding site to the *sre-1* promoter sequence in close proximity to the *sre-1* E-box motif likely does not recruit HLH-2 and HLH-3 to the *cis*-regulatory region of the *sre-1* chemoreceptor gene. Based on these findings, the question arises how introduction of the MEF-2 site into the *sre-1* promoter results in starvation-dependent regulation of *sre-1* without HlH-2/3. One possibility is that in response to starvation MEF-2 can repress *sre-1* through other bHLH factors that bind to the *sre-1* E-box, suggesting that MEF-2 may act as a state-dependent regulator more generally.

Our results on the *cis*-regulatory analysis and genetic experiments of *srh-234* suggest that MEF-2 may act as a transcriptional co-regulator for bHLH heterodimers in ADL neurons to temporarily regulate *srh-234* expression levels as a function of feeding state (Fig 7). In this model, feeding state-dependent expression changes in *srh-234* expression are directed by multiple transcriptional modules, where one module consists of a MEF-2 transcription factor and its MEF2 binding site, and the other module consists of a bHLH heterodimer (e.g. HLH-2/3, and HLH-2/4) and its bi-partite E-box binding site. When fed, MEF-2 activity is low and is prevented from repressing HLH-2/3/4 factors, and expression of *srh-234* in ADL is increased. When starved, MEF-2 is no longer inhibited, allowing binding to the MEF2 site, which in turn represses HLH-2/3/4-mediated activation of *srh-234* at the E-box site, ultimately leading to the decrease in *srh-234* expression.

### MXL-3/HLH-30 act non-cell-autonomously in the intestine to regulate *srh-234* expression

In addition to bHLH factors (i.e. HLH-2/3/4) and the MEF-2 transcription factor, we showed that both the TFEB ortholog HLH-30 and Max-like 3 homolog MXL-3 regulate chemoreceptor gene expression as a function of feeding state. Genetic epistasis experiments suggest that *mxl-3* and *hlh-30* act upstream of *mef-2*, *hlh-2* and *hlh-3* in the regulation of *srh-234* expression. Using cell-specific rescue and selective RNAi feeding experiments, we showed that the function of MXL-3 and HLH-30 is required, at least in part, in intestinal cells but not in ADL to regulate the expression of *srh-234* in ADL. In both *C*. *elegans* and mammals, HLH-30 TFEB coordinates a transcriptional program by regulating the expression of autophagy in response to nutritional stress [21,52], as well as the expression of host-defense genes [53]. Moreover, HLH-30 acts together with MXL-3 as a transcriptional switch to antagonistically regulate lysosomal lipase genes in the *C*. *elegans* intestine [21]. We find a similar antagonistic role for MXL-3 and HLH-30 acting in the intestine such that *srh-234* expression in ADL neurons is strongly enhanced in starved *hlh-30* mutants, while *mxl-3* mutants reduce *srh-234* expression levels in fed conditions. In contrast, in starved *mxl-3* mutants, *srh-234* expression is similarly reduced as in starved wild-type animals, suggesting that MXL-3 does not appear to regulate *srh-234* expression in response to starvation. How then may MXL-3 and HLH-30 activity regulate *srh-234* expression as a function of feeding state? Previous elegant work [21] showed that the transcriptional activity of *mxl-3* and *hlh-30* is highly regulated by feeding state conditions, such that upon starvation, *mxl-3* transcription in the intestine is rapidly down regulated, and at the same time, *hlh-30* transcription is induced in the *C*. *elegans* intestine. In this scenario, it is possible that in starved *mxl-3* mutants, HLH-30 activity is high and can therefore continue to repress and decrease *srh-234* expression to starved wild-type levels. Conversely, in starved *hlh-30* mutants, the loss in repression of *srh-234* expression mediated by HLH-30, turns on and increases *srh-234* expression to near fed wild-type levels. In order for this to happen, both MXL-3 and HLH-30 likely act antagonistically on the same E-box site of shared target genes, similar as proposed for lysosomal lipase gene regulation in the *C*. *elegans* intestine [21]. Taken together, these results suggest a non-cell-autonomous antagonistic role for MXL-3 and HLH-30 in the regulation of *srh-234* gene expression as a function of feeding state.

### An intestine-to-neuron model for regulation of *srh-234* as a function of feeding state

How might the action of bHLH factors (i.e. MXL-3 and HLH-30) in a remote tissue such as the intestine regulate the expression of a MEF-2 and bHLH-dependent chemoreceptor gene in the ADL neuron? Previous work has shown that communication between neurons and the intestine via insulin signaling is important for the regulation of metabolism, longevity and dauer formation of *C*. *elegans* [47,49,54]. The intestine, in particular, appears to be a major site for transcriptional regulation of insulin-like peptide (ILP) genes in response to feeding state conditions (fed vs starved) [48]. Our results reveal that insulin signaling from the intestine is similarly important for the regulation of *srh-234* expression in ADL. First, we show that *daf-2/daf-16* insulin signaling pathway acts downstream of *mxl-3* and *hlh-30* in the regulation of *srh-234*. Moreover, rescue experiments suggest that MXL-3 and HLH-30 act specifically in the intestine to remotely regulate *srh-234* expression in ADL. Second, MXL-3-mediated regulation of *srh-234* is dependent on DAF-2 signaling acting in ADL. Third, *mxl-3* mutants appear to alter the subcellular localization of DAF-16::GFP in ADL, suggesting that MXL-3 function is required to repress DAF-16 in ADL. Fourth, we show that starvation as well as reduced *mxl-3* and *daf-2* signaling increases the expression of *mef-2*, and this higher MEF-2 activity may repress the bHLH-mediated activation of *srh-234*. The specific mechanism by which DAF-2/DAF-16 signaling regulates MEF2 activity remains to be deciphered, but interestingly, the DAF-16 ortholog FOXO has been shown to work in concert with MEF2 family members during cardiovascular development [55]. Lastly, we show that *mxl-3* mutants reduce, albeit weakly, the expression of a candidate agonistic *ins-3* ILP in the intestine, which when specifically knocked down by RNAi in the intestine weakly reduces *srh-234* expression. Thus, these data suggest a non-cell-autonomous intestinal role for a *mxl-3*-dependent *ins-3* ILP in the regulation of *srh-234*.

It is likely that the *ins-3* ILP does not act individually on the DAF-2 pathway in ADL. We found that expression of *daf-2* in ADL neurons can rescue the reduced *srh-234* expression phenotype of *mxl-3; daf-2* double mutants (Fig 5C), suggesting that the *ins-3* ILP does not act as the limiting factor for activation of DAF-2 in ADL to regulate *srh-234* expression. Moreover, *ins-3* is also expressed in sensory neurons, ASI and ASJ, all of which likely do not express *mxl-3* [21], suggesting that *mxl-3*-independent mechanisms likely exist to drive *ins-3* expression. Consistent with these findings, *daf-2* mutants show a significantly stronger reduced *srh-234* expression than *mxl-3* mutants, suggesting that there must be additional factors that may regulate ILPs besides MXL-3. In addition to the *ins-3* ILP, we also found that other ILPs from the intestine (e.g. *ins-7*, *ins-21*, *ins-28*) as well as ILPs from other neurons (e.g. *ins-26* ASE) negatively regulate *srh-234* expression in fed conditions. The combined action of the ILPs from different tissues (the intestine) and neurons (e.g. ASE) on *srh-234* regulation is not yet clear. ILPs are known to regulate each other transcriptionally in complex networks in a combinatorial and coordinated fashion [54,56]. Moreover, a complex network of ILPs secreted from head neurons are known to both promote and oppose dauer entry via intestinal DAF-2 activity [47]. In this regard, perhaps ILPs secreted by the intestine and other neurons mediated by *mxl-3*-dependent and independent transcriptional mechanisms could both promote and antagonize DAF-2 activity in ADL to dynamically modulate *srh-234* expression as a function of feeding state.

### Concluding remarks

Plasticity in chemoreceptor gene expression may be a simple strategy by which an animal can modulate its chemosensory responses in changing external and internal state conditions. Our results describe cell-autonomous and non cell-autonomous transcriptional mechanisms involving MEF-2 and basic helix-loop-helix (bHLH) factors and their cognate binding sites, respectively, by which *C*. *elegans* may integrate and translate feeding-state information into proper expression changes of individual chemoreceptor genes. We expect that continued investigation of the mechanisms by which the expression of individual chemoreceptor genes are changed by internal state and external conditions such as food availability will lead to insights into the general principles of behavioral plasticity. Moreover, understanding chemoreceptor gene regulation could provide important insight into the mechanisms by which feeding-state dependent host-seeking and leaving behaviors in mosquitoes, and possibly parasitic nematodes, are accompanied by modulation of chemoreceptor gene expression.

## Materials and Methods

### Strains and culture

Worms were grown at 20°C under standard conditions on nematode growth medium (NGM) with *E*. *coli* OP50 as the primary food source as previously described [22, 57]. The wild-type strain used was *C*. *elegans* variety Bristol, strain N2. Mutant strains used in this study were: PD4605 *hlh-1(cc561)*, JK4099 *hlh-2(tm1768)*, OH9330 *hlh-3(ot354)*; *otIs73*, GD211 *rol-6(e187)hlh-6(tm299)unc-4(e120)*, RB747 *hlh-10(ok516)*, RB2177 *hlh-11(ok2944)*, VC1220 *hlh-25(ok1710)*, RB1337 *hlh-26(ok145)*, JIN1375 *hlh-30(tm1978)*, VC1904 *hlh-34(gk1031)*, CF1097 *ref-1(mu220)*, JK3276 *hnd-1(q740)*, VC1644 *cnd-1(gk781)*, MT372 *lin-22(n372)*, RB1588 *mxl-3(ok1947)*, KM134 *mef-2(gv1)*, CB1370 *daf-2(e1370)*, CF1038 *daf-16(mu86)*, QZ83 *daf-28(tm2308)*, PY10708, *ins-26(tm1983)*, HT1765 *unc-119(ed3)*; *wwEx77*[*ins-26p*::*gfp*, *unc-119(+)*], HT1690 *unc-119(ed3)*; *wwIs26*[*ins-3p*::*gfp*, *unc-119(+)*], HT1693 *unc-119(ed3)*; *wwExEx63*[*ins-4p*::*gfp*, *unc-119(+)*], HT1695 *unc-119(ed3)*; *wwEx64*[*ins-5p*::*gfp*, *unc-119(+)*], PY4108 *mef-2(gv1*); *Ex*[*mef-2p*::*mef-2*::*gfp*, *rol-6(su1006)*], TU3596 *sid-1(pk3321)him-5(e1490); lin-15B(n744)* and NL2099 *rrf-3(pk1426)*. Transgenic strains used in this study were: VDL3 *oyIs56*[*srh-234*p(165bp)::*gfp*, *unc-122*p::*rfp*], and *daf-2(e1307)*; *oyIs56*; *Ex*[*sre-1*p::*daf-2 cDNA*] [22], UL1583 *unc-119(ed3);IeEx1583*[*hlh-4*::*gfp*, *unc-119(+)*] and VL507 *unc-119(ed3); wwIs20*[*hlh-2*::*mCherry*::*his-11*, *unc-119(+)*] [20]. Double mutant strains were constructed using standard genetic methods, and the presence of each mutation was confirmed by PCR or sequencing.

### Measurement and quantification of *srh-234* and *sre-1 gfp-*reporter expression levels

Transgenic animals carrying promoter::*gfp* reporters were grown at 20°C on NGM plates seeded with *E*. *coli* OP50 as the bacterial food source. Young adult animals were washed with M9 buffer (to remove any bacteria in the gut) and transferred onto plates with *E*. *coli* OP50 bacterial food (fed) or without food for 12–24 hours (starved) unless indicated otherwise. Levels of promoter::*gfp* expression (e.g. *srh-234*, *sre-1*) were imaged and measured under a microscope equipped with epifluorescence as previously described [22]. For each promoter::*gfp* construct, expression from the same extra chromosomal array was examined in wild-type and mutants. Briefly, we mounted animals on agarose pads containing 3 mM sodium azide unless indicated otherwise, and visualized these on a Leica DM5500 compound microscope or a Leica TCS SP8 confocal equipped with epifluorescence. Microscope and camera settings were kept constant between images of different genotypes and conditions, unless indicated otherwise. The mean pixel intensity of *gfp* fluorescence in the entire cell-body of ADL was quantified using Volocity software. Prior to measurement, images of ADL were cropped for *srh-234* expression analysis. Statistical analyses of *srh-234* expression were performed using the non-parametric two-tail Mann-Whitney test.

### Expression constructs of *bHLH* genes and generation of transgenic animals

Expression vectors were generated by amplifying either the wild-type genomic sequences of *hlh-2*, *hlh-3*, *mxl-3*, or cDNA of *hlh-30* (this study), and cloned in a pSM *SL2*::*mCherry (or gfp)*::*unc-54* 3’UTR vector (a kind gift from Cori Bargmann) using cell- and tissue-specific promoters. This resulted in the generation of the expression constructs: pMG25 *sre-1*p(1 kb)::*mxl-3 genomic*::*SL2*::*mCherry* (*ADL*::*mxl-3*), pMG28 *ges-1*p(1.9 kb)::*mxl-3 genomic*::*SL2*::*mCherry* (*intestine*::*mxl-3*), pMG47 *mxl-3*p(2.1 kb)::*mxl-3 genomic*::*SL2*::*mCherry* (*mxl-3*p::*mxl-3*), *hlh-30*p(2.2 kb)::*hlh-30*::*SL2*::*mCherry* (*hlh-30*p::*hlh-30*), pMG65 *ges-1*p(1.9 kb)::*hlh-30* cDNA (*intestine*::*hlh-30*), pMG72 *sre-1*p(1 kb)::*hlh-30* cDNA (*ADL*::*hlh-30*), pMG30 *ges-1*p(1.9 kb)::*hlh-2 genomic*::*SL2*::*mCherry* (*intestine*::*hlh-2*), pMG73 *svh-1*p(1.8 kb)::*hlh-2 genomic*::*SL2*::*mCherry* (*ADL*::*hlh-2*), pMG71 *hlh-3*p(1.5 kb)::*hlh-3 genomic*::*SL2*::*gfp (hlh-3p*::*hlh-3*::*SL2*::*gfp)*, pJG17 *sre-1*p(1 kb)::*daf-16a*::*gfp* (*ADL*::*daf-16*::*gfp*], and pVDL20 *str-148*p(1.6 kb)::*mxl-3 genomic*::*SL2*::*mCherry* (*AWC*::*mxl-3*). The construct *ges-1p*::*mxl-3IR* (plasmid 249/250) used for knocking down the *mxl-3* gene specifically in the intestine via RNAi (*intestine*::*mxl-3 RNAi*) is a kind gift from Eyleen O’Rourke [21], and the ADL-specific reporter *sri-51*p(3 kb)::*mCherry* was a kind gift from Cori Bargmann [12].

For generating the *hlh-10*p::*gfp* and *svh-1*p::*gfp* reporters, we fused about 1.5 kb and 1.6 kb promoter sequence of *hlh-10* and *svh-1*, respectively, to *gfp* as previously described [58]. The *sre-1*p::*gfp* construct was generated by inserting about 1 kb promoter sequence of *sre-1* into the pPD95.77 vector (AddGene). Transgenic animals were generated using either the *unc-122*p::*dsRed* (50–100 ng/μl) or the pRF4 *rol-6(su1006)* co-injection markers injected at 150 ng/μl using a standard microinjection protocol [59]. Expression constructs were injected at 20–30 ng/μl. All amplified products in the generated constructs were sequenced to confirm the absence of errors generated via the amplification procedure.

For co-expression analysis of *bHLH genes*, we crossed either transgenic lines of *hlh-2*::*mCherry*::*his-11* (strain VL507) [20], *hlh-4*p::*gfp* (strain UL1583) [20], and *hlh-3*p(1.5 kb)::*hlh-3 genomic*::*SL2*::*gfp* (this study) with transgenic lines of the relevant ADL-specific transgenic reporter lines, either *sri-51*p(3 kb)::*gfp* or *srh-234*p(165 bp)::*gfp*,

### Examination of the *cis*-regulatory region of *srh-234*

Searching the 165 bp upstream sequence of *srh-234* for transcription factor binding sites using Lasagna 2.0 (http://biogrid-lasagna.engr.uconn.edu/lasagna_search/) [31] identified a candidate E-box motif (CANNTG) and a MEF2 site (CTA(A/T)4TA(G/A)) [33]. A *srh-234*p::*gfp* reporter was generated to create site-directed mutations in the candidate binding sites. For this, we cloned 165 bp upstream sequence of the predicted *srh-234* translational start codon into the *Hind*III and *Bam*HI sites of the pPD95.77 vector (Addgene). The resulting *srh-234*p*(WT)*::*gfp* construct was used to perform site-directed mutations in the E-box and MEF2 binding sites with help of the QuickChange II site-directed mutagenesis kit (Agilent Technologies) to generate the following promoter::*gfp* constructs: *srh-234*p*(-E-box)*::*gfp*, *srh-234*p*(-E-box L)*::*gfp*, *srh-234*p*(-E-box R)*::*gfp*, *srh-234*p*(-MEF2)*::*gfp*, and *srh-234*p*(-E-box/MEF2)*::*gfp*. For generating the *sre-1*p*(+MEF2)*::*gfp* construct, we inserted the extended *srh-234* MEF2 site (AATAGTTATATTTAAAAA) at position -100 bp into 1.1 kb promoter sequence of *sre-1* using the Q5 site-directed mutagenesis kit (New England Biolabs). The same mutagenesis kit was used to generate the *odr-10*p*(+E-box)*::*gfp* construct by inserting the extended *srh-234* E-box motif (AATCACCTGTCC) into the *odr-10* promoter sequence at position -70 bp. All created site-directed mutations were confirmed by sequencing. Transgenic animals of promoter::*gfp* constructs were generated in wild-type animals using a standard microinjection protocol [59]. Promoter::*gfp* constructs were injected at 30 ng/μl together with either the pRF4 *rol-6(su1006)* or *unc-122*p::*dsRed* as co-injection markers at 150 ng/μl.

### ADL-mediated avoidance assays

For examining avoidance responses to CuCl_2_, glycerol and SDS, we used the drop-test assay as previously described [60,61]. Briefly, we picked L4-staged larvae of either wild-type, *hlh-2(tm1768)* or *hlh-3(ot354)* mutants about 20 hours before the assay to NGM plates with (on-food) or without food (off-food). For on-food plates, 25 μl of overnight culture of *E*. *coli* OP50 in LB was spread on each plate. For off-food plates, 25 μl of LB was spread on each plate. All plates were allowed to dry for 1 hour without lids. Animals were picked using an eyelash pick to a plate without food for less than a min (to prevent food being transferred), before animals were transferred to on-food and off-food assay plates. Animals were allowed to settle for 10 min, and then assayed using a capillary to deliver the repellent drop. About 5 animals were assayed on each plate at room temperature, all animals were stimulated every 60 seconds and the percentage of responding animals were recorded. The avoidance index is the number of positive responses divided by the total number of trials. The tested repellents were 2 mM CuCl_2_ (Acros Organics), 0.5 M glycerol (Sigma) and 0.1% SDS (Thermo Scientific). All repellents were dissolved and diluted in M13 buffer (30 mM Tris, 100 mM NaCl, 10 mM KCl).

For examining the avoidance response to octanol, we used the smell-on-a-stick assay as previously described [62,63]. Briefly, the blunt end of a paintbrush hair (Loew-Cornell, 9000 Kolinsky 7 size) was taped to a Pasteur pipette, and dipped in either 30% or 100% 1-octanol and placed in front of either a forward-moving wild-type, *hlh-2(tm1768)* or *hlh-3(ot354)* mutant animal. The time to respond (seconds) is the amount of time it took for the animal to initiate backward movement at room temperature. Octanol (Sigma) was diluted and freshly made in ethanol.

### RNAi feeding of *bHLH* genes

Feeding RNAi of selected *bHLH* genes was performed in the *rrf-3(ok1426)* background to enhance neuronal RNAi [64]. Briefly, L4-staged larvae of *rrf-3(pk1426)* mutant animals carrying a stably integrated *srh-234p*::*gfp* transgene (*oyIs56*) were placed on an NGM agar plate containing 1 mM IPTG and seeded with *E*. *coli* HT115 bacterial food producing dsRNA directed against the selected *bHLH* genes. Bacteria expressing dsRNAs directed against selected *bHLH* genes were taken from the *C*. *elegans* feeding RNAi library (Source BioScience). For *hlh-2(RNAi)* and *hlh-4(RNAi)*, we cloned the wild-type genomic sequence of *hlh-2* and *hlh-4* in the L4440 vector (Addgene) because these bacterial RNAi clones in the feeding RNAi library were incorrect. For fed conditions, animals fed with *bHLH(RNAi)* were scored as adults in the next generation (F1), and expression of *srh-234*p::*gfp* was measured and quantified as described above. For starved conditions, animals in the F1 generation were transferred as L4-staged larvae to RNAi plates without food and *srh-234*p::*gfp* expression was determined about 12 hours later. The L4440 empty vector was used as the RNAi control. All RNAi bacterial clones used were cultured as previously described [65], and confirmed by sequencing.

### Tissue-specific RNAi of *bHLH* and *ILP* genes

For tissue-selective knockdown of *bHLH* and *ILP* genes, we employed a previously described feeding RNAi strategy [38]. Briefly, we amplified and cloned the wild-type genomic sequence of *sid-1* in the pSM *SL2*::*mCherry*::*unc-54* 3’UTR vector (a kind gift from Cori Bargmann) using the ADL-specific promoter, *sre-1*, and the intestinal-specific promoter, *ges-1*, resulting in constructs: pMG57 *sre-1*p::*sid-1 genomic*::*SL2*::*mCherry* (*ADL*::*sid-1*), and pMG66 *ges-1*p::*sid-1 genomic*::*SL2*::*mCherry* (*intestine*::*sid-1*). Transgenic animals were generated in *sid-1(pk3321)* mutant animals carrying the stably integrated *srh-234*p::*gfp* transgene (*oyIs56*) using a standard microinjection protocol [59]. Expression constructs were injected at 30 ng/μl together with the pRF4 *rol-6(su1006)* as a co-injection marker at 150 ng/μl. To selectively enhance RNAi in either ADL neurons or in the intestine (but not in other tissues), L4-staged larvae of transgenic animals carrying either *ADL*::*sid-1* or *intestine*::*sid-1* were placed on an NGM agar plate containing 1 mM IPTG and freshly seeded with *E*. *coli* HT115 bacterial food producing dsRNA directed against individual *bHLH* genes or *ILP* genes as previously described [65]. Expression of *srh-234*p::*gfp* was measured and quantified in transgenic animals fed with either *bHLH(RNAi)* or *ILP(RNAi)* in the next generation (F1) at the adult stage unless indicated otherwise. The L4440 empty vector was used as the RNAi control, and all RNAi bacterial clones used were confirmed by sequencing.

### Subcellular localization of DAF-16::GFP

For measuring the fraction of DAF-16::GFP in the compartment (cytoplasmic:nuclear) of ADL, we generated transgenic animals that carry both a *sre-1*p::*daf-16a*::*gfp* reporter as an extra chromosomal array (*ADL*::*daf-16*::*gfp*] and the integrated *hlh-2*p::*mCherry*::*his* reporter. Transgenic fed animals were grown at room temperature until young adults, and then transferred onto plates with *E*. *coli* OP50 bacterial food (fed) or without food for 12 hours (starved). Next, animals were mounted on glass slides containing 1 mM levamisol to paralyze the animals, and visualized as described above. Fluorescent images were taken at the same exposure time using Volocity software. The fraction of DAF-16::GFP in the subcellular compartment of ADL neurons was measured by counting the fraction of animals that show nuclear fluorescence in either the red (cytoplasm > nuclear), orange (nucleus = cytoplasm), or yellow color (nucleus > cytoplasm). Statistical analyses of the distribution between genotypes and conditions was performed using the χ^2^ test.

### qRT PCR of *srh-234* message

As described previously [22], total RNA was isolated from a growth-synchronized population of adult animals and reverse transcribed using oligo(dT) primers. Real-time quantitative reverse transcription–PCR (qRT–PCR) was performed with a Biorad C1000 Touch Thermal Cycler, Platinum Taq polymerase (Invitrogen), and primers specific for *srh-234* and *odr-10* coding sequences. Primer sequences for *srh-234* are 5′-GGACAATTGAAATGCAACACA-3′ and 5′-GACGGGGACAATAAAGAGCA-3′. Primer sequences for *odr-10* are 5′-GAGAATTGTGGATTACCCTAG-3′ and 5′-CTCAATATGCATTATAGGTCGTAATATG-3′.

### Dye-filling of ADL sensory neurons

Dye-filling experiments were performed similarly as previously described [66]. A stock dye solution containing 5 mg/μl red fluorescent lipophilic dye DiI (Sigma Aldrich) was diluted in M9 buffer by 10,000 times for optimal signal intensity. Animals were soaked in the fluorescent dye solution for one hour and then rinsed with M9 buffer twice. Stained animals were recovered for 1 hour on NGM plates seeded with *E*. *coli* OP50 bacterial food before examination of ADL sensory neurons.

## Supporting Information

S1 Fig The *odr-10* promoter with the *srh-234* E-box motif is expressed in AWA and ADL sensory neurons. **(A)** The indicated length and position of the inserted E-box motif of *srh-234* relative to the translational start site of *odr-10* fused to *gfp* in expression vectors (see Material and Methods). **(B)** Representative expression of AWA and ADL sensory neurons driven by the *odr-10*p*(+E-box)*::*gfp* transgene in well-fed adults. Anterior is left. Ventral view of images were acquired at the same exposure time at room temperature. **(C)** Relative expression of *odr-10p(+E-box)*::*gfp* in adult *hlh-2* mutants. Data shown is for one transgenic line with n>20 adult animals. ** indicates values that are different from that of wild-type adult animals at *P*<0.05. Error bars denote the SEM.
(EPS)

S2 Fig Analysis of *bHLH* genes on *srh-234* expression and dye-filling of ADL. **(A)** Relative expression of *srh-234*p::*gfp* in the ADL cell body of adults of the indicated genotypes during well-fed and starved conditions. >20 adult animals for each genotype and condition. **(B)** Relative expression of *srh-234*p::*gfp* in adult animals fed with RNAi directed against *bHLH* genes. Feeding RNAi was performed in *rrf-3(pk1426)* RNAi hypersensitive mutant animals (see Material and Methods). **(C)** Data shown is the ratio of endogenous *srh-234* message to endogenous *odr-10* message as quantified by qRT–PCR in adult wild-type and *mxl-3* mutants when fed or starved. Expression of *odr-10* is unaffected by starvation in hermaphrodites. The mean of the ratios from two independent experiments is shown. * indicates values that are different from fed wild-type animals at *P*<0.01 using a two-sample *t*-test. **(D, E)** Percentage of adult mutant animals **(D)** and RNAi-treated animals **(E)** of the indicated genotypes showing normal ADL dye-filling compared to wild-type (see Material and Methods). n>20 adult animals for each genotype. * and ** indicates values that are different from that of wild-type adult animals at *P*<0.001, and *P*<0.05, respectively. Error bars denote the SEM. **(F)** Summary of the main bHLH factors that regulate *srh-234* expression without altering ADL neuron identity. Proteins in parentheses denote minor bHLH factors that negatively or positively regulate *srh-234* expression.
(EPS)

S3 Fig *hlh-2* and *hlh-3* mutants show wild-type avoidance responses to known ADL-mediated chemical cues. **(A)** Avoidance responses of adult wild-type, *hlh-2* and *hlh-3* mutants to CuCl_2_, glycerol and SDS at the indicated concentrations. Drop-test assays were performed at room temperature (see Material and Methods). n>70 trials for each genotype. # indicates values that are different between the genotypes compared by brackets at *P*<0.05. (**B)** Response (reversal) time of adult wild-type, *hlh-2* and *hlh-3* mutants to either 100% or 30% 1-octanol. Smell-on-stick assays were performed at room temperature (see Material and Methods). n>20 trials for each genotype. In all experiments, error bars denote the SEM, and ns indicates not significantly different.
(EPS)

S4 Fig Expression of *sre-1* in *bHLH* mutants. **(A)** The indicated lengths and positions of predicted regulatory elements relative to the translational start site of *sre-1* fused to *gfp* in expression vectors (see Material and Methods). The sequence in blue indicates the predicted endogenous E-box motif of *sre-1*. **(B)** Representative expression of ADL sensory neurons driven by wild-type *sre-1* regulatory sequences in adult wild-type and mutant animals of the indicated *bHLH* genes in well-fed conditions. Images are lateral views of ADL sensory neurons with the arrow pointing to the cell body; anterior is left. Images were acquired at the same exposure time at room temperature. The same *sre-1*p::*gfp* transgene was examined in adult wild-type and mutant animals. **(C, D)** Relative expression of wild-type *sre-1*p::*gfp* **(C)** and *sre-1p(+MEF2)*::*gfp* **(D)** in mutants of *bHLH* genes compared to wild-type adults when fed or starved. n>25 adult animals for each genotype. Error bars denote the SEM.
(EPS)

S5 Fig Expression of *svh-1* in ADL neurons is not altered in the hypomorphic *hlh-2* mutant. **(A)** The indicated length and position of a predicted endogenous E-box motif of *svh-1* relative to the translational start site of *svh-1* fused to *gfp* in expression vectors (see Material and Methods). **(B)** Representative expression of ADL sensory neurons (arrow) driven by the *svh-1*p::*gfp* transgene in well-fed adult animals. The same *svh-1p*::*gfp* transgene was examined in adult wild-type and mutant animals. Anterior is left. Ventral view of images was acquired at the same exposure time and at room temperature.
(EPS)

S6 Fig Co-expression analysis of *bHLH* genes in ADL during development. **(A-C)** Expression of *hlh-2* **(A)** *hlh-3* **(B)** or *hlh-10* **(C)** with either *sri-51*p::*mCherry* or *srh-234*p::*gfp* as ADL-specific reporters in larvae and embryos. ADL neurons are indicated with arrows. When co-expressed, ADL neurons are shown as a yellow cell in a merged fluorescent image. For animals carrying the *hlh-2p*::*mCherry*::*his-11* transgene expressing *hlh-2* in the nucleus ADL appears with a green cytoplasm and yellow nucleus in a merged fluorescent image. int, intestine; ph, pharynx.
(EPS)

S7 Fig Selective down regulation of *bHLH* genes in ADL and the intestine by RNAi. **(A)** Overview of tissue-specific knockdown of *bHLH* genes by RNAi. (**B-C)** Representative expression of *srh-234*p::*gfp* in adult animals by feeding either *hlh-2(RNAi)*, *hlh-3(RNAi)*, *hlh-4(RNAi)*, *mxl-3(RNAi)* or control RNAi. Feeding RNAi was performed in *sid-1(pk3321)him-5(e1409)* mutants with the stably integrated *oyIs56* transgene carrying either a *ADL*::*sid-1* **(B)** or *intestine*::*sid-1* **(C)** extra chromosomal transgene to selectively enhance RNAi in ADL neurons or in the intestine, respectively, but not in other tissues (see Material and Methods). Images are lateral views of ADL with the arrow pointing to the cell body; anterior is left. Images were acquired at the same exposure time at room temperature. **(D-E)** Relative expression of *srh-234*p::*gfp* in the ADL cell body of adult animals when fed either *hlh-2(RNAi)*, *hlh-3(RNAi)*, *hlh-4(RNAi)*, or *mxl-3(RNAi)* compared to adult animals fed with control RNAi. Data shown is the average of at least two independent transgenic lines with n>20 adult animals for each line. * indicates values that are different from that of wild-type adult animals at *P*<0.001, and # between the genotypes compared by brackets at *P*<0.001. Error bars denote the SEM.
(EPS)

S8 Fig Expression of *mef-2* and *hlh-2* in different mutants and feeding state conditions. **(A)** Representative expression of *mef-2*::*mef-2*::*gfp* in adult wild-type animals when fed and starved (upper panels), as well as adult *daf-2* and *mxl-3* mutants (lower panels). **(B)** Representative expression of *hlh-2*::*mCherry*::*his-11* in adult wild-type animals when fed and starved (upper panels) and adult *daf-2* and *daf-16* mutants (lower panels). In all experiments, images were taken at the same exposure time at room temperature. Anterior is right. The same transgene was examined in each genotype and condition.
(EPS)

S9 Fig Selective down regulation of *ILP* genes in the intestine by RNAi. **(A)** Relative expression of *srh-234*p::*gfp* in the ADL cell body of adult animals by feeding individual *ILP(RNAi****)*** compared to adult animals fed with control RNAi. Feeding RNAi was performed in *sid-1(pk3321)him-5(e1409)* mutants with the stably integrated *oyIs56* transgene carrying the *intestine*::*sid-1* extra chromosomal transgene to selectively enhance RNAi in the intestine, but not in other tissues (see Material and Methods). Data shown is the average of at least two independent transgenic lines with n>20 adult animals for each line. * and ** indicates values that are different from that of wild-type adult animals at *P*<0.001 and *P*<0.05, respectively. Error bars denote the SEM. **(B)** Relative expression of *srh-234*p::*gfp* in ADL neurons of adult *daf-28*, *ins-26* and *mxl-3* mutants (ILPs) in well-fed conditions. n>25 adult animals for the indicated genotypes. Error bars denote the SEM. **(C)** Images are lateral views of ADL with the arrow pointing to the cell body. Anterior is left. Images were acquired at the same exposure time at room temperature. **(D)** Summary of the main insulin-like peptides (ILPs) that regulate *srh-234* expression in ADL. Proteins in parentheses denote minor ILPs that negatively or positively regulate *srh-234* expression.
(EPS)

S10 Fig Expression patterns of *ILP* genes in *mxl-3* mutants. **(A-C)** Representative expression of selected *ILP* genes *ins-3*p::*gfp* **(A)**, *ins-4*p::*gfp* and *ins-5*p::*gfp* **(B)** and *ins-26*p::*gfp* **(C)** in adult wild-type (left panels) and *mxl-3* mutant animals (right panels) during fed conditions. White box represents intestinal area used for comparative analysis of expression. In all experiments, images were taken at the same exposure time at room temperature. The same transgene was examined in each genotype. Arrow indicates ASE neuron.
(EPS)

S1 Table Predicted MXL-3 and HLH-30 binding sites in insulin-like peptide promoters. A search of possible MXL-3 and HLH-3 binding sites present in promoter sequences of insulin-like peptide genes using Lasagna 2.0 (http://biogrid-lasagna.engr.uconn.edu/lasagna_search/) with a cut-off value of *P*<0.01.
(XLSX)
